# Take the reins: a study protocol of a randomized controlled trial testing the effects of time-restricted eating vs. nutrition control on cancer-related fatigue among survivors of hematological malignancies

**DOI:** 10.1186/s40795-025-01185-0

**Published:** 2025-10-31

**Authors:** Amber S. Kleckner, Carin L. Clingan, Ashraf Z. Badros, Emily N. C. Manoogian, Karen M. Mustian, Satchidananda Panda, Alice S. Ryan, Shijun Zhu

**Affiliations:** 1 Department of Pain and Translational Symptom Science, University of Maryland School of Nursing, Baltimore, MD USA; 2 Marlene and Claude Greenebaum Comprehensive Cancer Center, University of Maryland Medical Center, Baltimore, MD USA; 3 Department of Medicine, University of Maryland School of Medicine, Baltimore, MD USA; 4 Regulatory Biology, The Salk Institute for Biological Studies, La Jolla, CA USA; 5 Division of Supportive Care in Cancer, University of Rochester Medical Center, Rochester, NY USA; 6 Department of Veterans Affairs, Veterans Affairs Maryland Health Care System, Baltimore, MD USA; 7 Department of Organizational Systems and Adult Health, University of Maryland School of Nursing, Baltimore, MD USA

**Keywords:** Intermittent fasting, Diet, Nursing, Supportive care, Blood cancer, Quality of life

## Abstract

**Background:**

Cancer-related fatigue is one of the most common and debilitating side effects of cancer and its treatments. Fatigue may stem from disruptions in circadian rhythms and dysregulation of glucose metabolism, which can be improved through time-restricted eating. Time-restricted eating is a daily eating pattern that entails consuming food within a defined, consistent window (e.g., 10 h) every day. When the eating window aligns with the daylight hours, it can entrain circadian processes and modulate physiological regulation of whole-body metabolism. It is hypothesized that time-restricted eating can relieve cancer-related fatigue in blood cancer survivors via regulating circadian rhythms and improving metabolism.

**Methods:**

This trial is a phase II randomized controlled trial comparing the effects of time-restricted eating (10 h daytime feeding/14 h fasting at night) vs. a time-, attention, and expectancy-matched control nutrition counseling intervention (no time component) on cancer-related fatigue. A total of 96 blood cancer survivors will be recruited; eligible survivors will be 2 months to 2 years post-adjuvant chemotherapy, report moderate to severe fatigue, consume food within a window that is > 10 h, and not be employed in night-shift work. At baseline, 6 weeks, and 12 weeks (post-intervention), assessments include patient-reported fatigue, measures of circadian rest-activity rhythm, and glucose metabolism via continuous glucose monitoring. Participants will log their food intake using the myCircadianClock smartphone app at baseline and throughout the 12-week study. At 24 weeks (12 weeks post-intervention), questionnaires will probe maintenance of the dietary pattern and sustainability of any intervention effects on fatigue.

**Discussion:**

Time-restricted eating is scalable and free-of-cost, lending itself to accessibility for the vast majority of cancer survivors. Data generated herein will be used to inform a larger, phase III multisite clinical trial testing the effects of time-restricted eating on cancer-related fatigue among survivors of hematological malignancies, and further optimize interventions that entrain circadian rhythms and improve glucose metabolism to alleviate cancer-related fatigue and other supportive care outcomes.

**Trial registration:**

clinicaltrials.gov ID: NCT06482515.

**Supplementary Information:**

The online version contains supplementary material available at 10.1186/s40795-025-01185-0.

## Background

Cancer-related fatigue is a highly prevalent and persistent side effect of cancer treatment. It affects at least 30–90% of patients with risk factors including younger age, type of treatment (chemotherapy and radiation invoke worse fatigue than surgery), higher body mass index, and higher baseline depression symptoms [1–3]. Its severity can greatly hinder the ability to perform activities of daily living; prevent people from returning to work, family roles, and hobbies; and decrease quality of life [4]. The mechanisms underlying the etiology and pathophysiology of cancer-related fatigue seem to be related in part to inflammation, hypothalamic–pituitary–adrenal (HPA) activation dysfunction, metabolic and/or endocrine dysregulation, or other mechanisms, but are largely not understood, thereby thwarting the development of effective preventative strategies and treatments [5, 6].

Circadian rhythms are biological diurnal cycles that work in synchrony to regulate hormone secretion, the sleep/wake cycle, core body temperature, and other processes. Circadian rhythms and human health have a bidirectional relationship, and circadian disruption is associated with a broad range of pathologies including fatigue [7–9]. Circadian regulation occurs via genetic and physiological processes and is important to maintain homeostasis of the endocrine system, autonomic nervous system, and nutrient metabolism [10, 11]. The importance of maintaining circadian rhythms is clear when traveling across time zones, and “jet lag” is a common experience of fatigue when the circadian clock is disrupted [10]. Chemotherapy treatment disrupts circadian rhythms in regard to clock gene regulation [12], rest-activity rhythms [13], diurnal hormone secretion [14], etc., and changes in circadian rhythms are associated with fatigue [9, 15].

Regulation of circadian rhythms relies heavily on regular sleep habits and interventions to regulate circadian clock currently include bright light therapy, exercise, melatonin supplementation, and more recently, nutrient timing [10]. Indeed, there are consistent animal and human data demonstrating that aberrant feeding/eating patterns dysregulate objective circadian clock measures (i.e., expression of genes that show strong diurnal oscillations) [16]. In an observational study among 156 healthy American adults in California, Gill and Panda [17] showed that approximately 50% of people eat within a window greater than 14.75 h per day; only 10% eat within a window 12 h or less [11]. Similar results were observed in our pilot feasibility trial—out of 60 people expressing interest in the trial, only 5 (8%) already ate within a 10-h window and were therefore not eligible [18]. A consistent, shorter window of eating, for example 10 h or shorter, may aid in the regulation of the circadian clock and improve metabolic homeostasis with broad health outcomes [11, 19, 20]. Restricting eating to certain time window is called “time-restricted eating” (TRE).

TRE is a promising field of study and has gained traction in the media and the public over the past decade for its ability to strengthen circadian rhythms and improve health [19–21]. TRE involves restricting the consumption of calories to a short window (4–12 h) during normal waking hours, for example 8am-6pm; water is never restricted. Human (time-restricted *eating*) and rodent (time-restricted *feeding*) studies have shown that TRE helps to maintain metabolic homeostasis and, as a result, helps with weight management, improves sleep, and attenuates age- and diet-induced heart disease (review [20]). Hatori et al. clearly demonstrated the effects of nutrient timing on metabolic regulation: time-restricted feeding vs. time-*un*restricted feeding of mice led to weight loss despite equal energy intake [22]. In humans, Gabel et al. performed a single-arm 8-h TRE study among obese adults for 12 weeks (*n* = 23) [23]. Despite the diet being *ad libitum*, caloric intake decreased 341 ± 53 kcal/day; body weight decreased 2.6 ± 0.5%, and systolic blood pressure decreased 7 ± 2 mmHg. However, there were no significant changes in body composition, circulated lipids, fasting blood glucose, or fasting insulin. Also, Wilkinson et al. performed a single-arm 10-h TRE study among patients with metabolic syndrome for 12 weeks (*n* = 19) [11]. They observed improvements in sleep efficiency and quality, a safe rate of decrease in body weight and body fat percentage, a reduction in total and low density lipoprotein (LDL) cholesterol, and reductions in systolic and diastolic blood pressure [11]. While these studies were implemented for 12 weeks, benefits of an 8-hour TRE window have been seen in glucose and lipid metabolism and circadian clock gene expression in as short as 4 days [24].

We targeted enrollment to survivors of hematological malignancies given the high prevalence of persistent fatigue (58% [1]) and high interference of fatigue with daily activities in this population [25, 26]. Hematological malignancies include leukemia, lymphoma, and multiple myeloma. While some blood cancers are curable, many are not. Advances in therapies, including targeted treatments, immunotherapy, and stem cell transplants, are extending life expectancy and improving quality of life for many patients who are living with blood cancer as a chronic condition. In fact, five-year survival rates tend to be over 67% and as high as 89% for Hodgkin Lymphoma [27–30]. Given the many years and decades that fatigue could reduce the quality of life of blood cancer survivors, treatments for fatigue could make a large positive impact.

To date, there been only several studies testing TRE in the cancer population including single-arm studies (e.g., [18, 31–33]), and our pilot randomized controlled trial (RTC) [34]; these studies have collectively shown that a 10-h TRE window is feasible, safe, and effective at improving metabolic markers, with potential to improve fatigue. Building upon this evidence, we are evaluating TRE vs. a time-, attention, and expectancy-matched control arm in an RCT with the ultimate goal of evaluating its effectiveness to treat cancer-related fatigue. While there are several TRE RCTs underway with fatigue as an outcome (e.g., [35, 36]), this is the only study to our knowledge with fatigue as the primary outcome.

## Methods

### Study overview

#### Study design

This is a phase II, parallel-group, RCT testing the effects of TRE vs. a nutrition control on cancer-related fatigue. Funded by the National Cancer Institute (NCI; R01CA284082), the trial is being conducted through the University of Maryland Medical System (NCT06482515, registered June 25, 2024). The primary aims are to provide initial estimates of efficacy of a 12-week TRE intervention vs. an unrestricted eating pattern on patient-reported fatigue among survivors of hematological malignancies experiencing fatigue. A secondary aim is to examine the sustainability of TRE and its effects on fatigue at 24 weeks. Mechanistic aims will assess the effects of TRE vs. control on circadian rest-activity rhythms and glucose metabolism, as well as assess associations between these measures. The study’s name is “Take the Reins,” which reflects how changing one’s diet can empower someone to control at least one aspect of their life that may have direct effects on their symptoms and healing.

#### Recruitment sites

Participants will be recruited from an academic medical center, University of Maryland Marlene and Stewert Greenebaum Comprehensive Cancer Center (UMGCCC), and community oncologist clinics associated with the University of Maryland Medical System in the United States. The community sites include the Cancer Institute at University of Maryland St. Joseph Medical Center in Towson, Maryland; the Tate Cancer Center at University of Maryland Baltimore Washington Medical Center in Glen Burnie, Maryland; the Kaufman Cancer Center at University of Maryland Upper Chesapeake Medical Center in Bel Air, Maryland; and the Cancer Center at University of Maryland Shore Regional Health in Easton, Maryland.

#### Study oversight

The research protocol was reviewed and approved by the University of Maryland Institutional Review Board (IRB; HP-00110284) and is being conducted in accordance with the Declaration of Helsinki. The Data and Safety Monitoring/Quality Assurance Committee (DSM/QAC) at the UMGCCC declared this trial “Minimal Risk,” and is overseeing the trial’s recruitment and safety at least twice per year. The DSM/QAC is comprised of clinicians, scientists, and community members affiliated with UMGCCC and is independent of NCI, the sponsor. There are not any interim analyses planned because cancer-related fatigue is not a life-threatening illness and both our nutrition interventions are safe; it is extremely unlikely that recruitment will need to close early.

### Eligibility criteria

Inclusion criteria (*Participants must*…):

- Have a diagnosis of a hematologic neoplasm (e.g., leukemia, lymphoma, multiple myeloma);
- Be at least 2 months post-treatment with chemotherapy, radiation, targeted therapy, chimeric antigen receptor (CAR)-T cell therapy, stem cell transplant, or another therapy (maintenance therapies are acceptable; steady unchanged treatment for relapsed disease for > 2 months and expected to stay on the therapy until progression is also acceptable);
- Have a baseline level of fatigue, as determined by at least one of the following:
  - Reporting a score of 4 or higher in response to the question, “What was your worst fatigue in the last week, on a scale of 0–10, where 0 is no fatigue and 10 is the worst fatigue?”
  - In the habit of taking daytime naps,
  - Have fatigue that interferes with their ability to work, engage in social events, or is more than would be expected from physical exertion,
- Be able to speak and/or read and write in English or Spanish;
- Be at least 18 years old; and
- Be able to provide informed consent.

Exclusion criteria (participants must not…)

- Be underweight, as defined as a body mass index < 18.5 kg/m^2^;
- Already eat all their food within a window that is 10 h or shorter most (6/7) days of the week (for this criterion, we ask when they typically have their first beverage or bite of breakfast in the morning, and when they typically have their last meal, snack, or beverage before bed; if this window is > 10 h the person is eligible; people who often eat during sleeping hours are also eligible);
- Be employed in a job where they regularly work away from the home at night (e.g., night shift);
- Have surgery planned during the study duration;
- Have any contraindications to the proposed nutrition intervention as identified by their medical provider, their designee, or the study team (e.g., type 1 diabetes, risk for hypoglycemia, medication requirements, pregnancy or plans for pregnancy, breastfeeding, recent history of an eating disorder);
- Be taking insulin; or
- Be on enteral or parenteral nutrition.

While taking part in this study, participants may concomitantly be part of drug trials or observational trials but may not be part of other trials that are testing behavioral interventions.

### Procedures (Fig. 1)

**Fig. 1:**
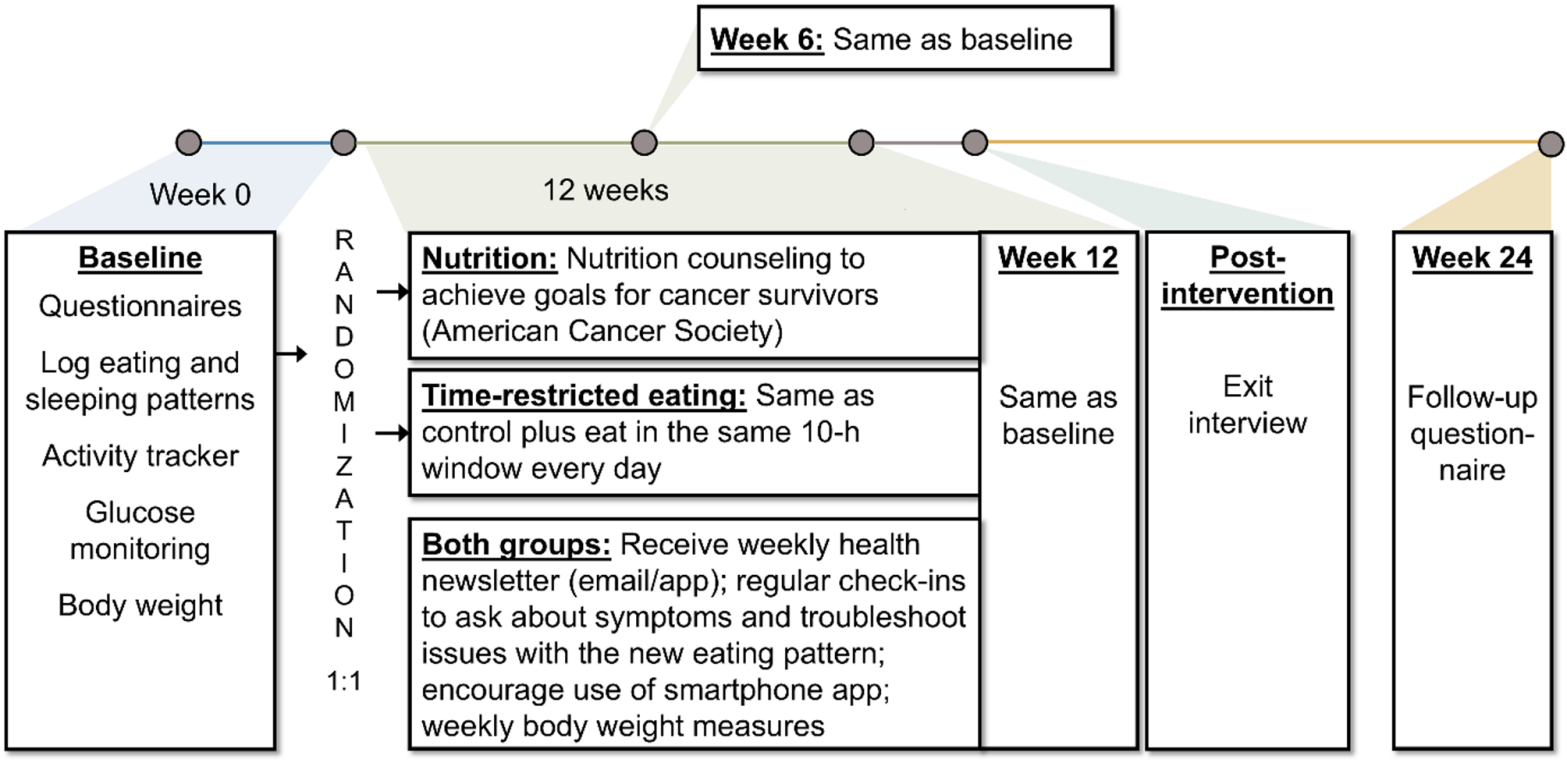
Study schema

All participants will provide written consent to participate in this trial. The consent document was developed using the template from the University of Maryland Baltimore’s Research Compliance Office. Participants are being consented to the study either on paper or electronically via Research Electronic Data Capture (REDCap) tools hosted at University of Maryland Baltimore [37, 38] by a member of the study team. After consent, participants are in the study for approximately 26 weeks. During the baseline week (7 days), participants are asked to complete questionnaires that probe fatigue (e.g., [39]), behaviors that correlate with fatigue (e.g., physical activity [40]), and related symptoms; log their food with time stamps via the myCircadianClock smartphone application (Salk Institute, La Jolla, CA, USA [41]); wear an actigraphy watch (MotionWatch8, CamNTech, Boerne, TX, USA); and wear a continuous glucose monitor (FreeStyle Libre, Abbott Nutrition, Chicago, IL, USA). Participants then return the actigraphy watch and glucose monitor reader to the lab via a postage-paid USPS box for data extraction. After baseline assessments, participants are randomized 1:1 to the TRE group (intervention) or the unrestricted eating group (control). The randomization table was generated in block sizes of 2 and 4 by the statistician (S. Zhu) and concealed from the other study team members using REDCap; there is no stratification. After baseline, all participants meet with a licensed dietitian nutritionist (e.g., C. Clingan) for individualized nutrition counseling over Zoom (Zoom Communications, San Jose, CA) by default, or in person or over the phone if desired. Due to the nature of the intervention, participants cannot be blinded. Those assigned to the TRE group are asked to meet recommendations within a consistent self-selected 10-hour eating window while those in the control group are not given temporal restrictions. Participants are asked to follow the recommendations for 12 weeks. A study team member (preferably the nutritionist/interventionalist) checks in with each participant via phone or Zoom approximately every 2 weeks. The same data collection protocol is conducted at week 6 and week 12. At week 24, questionnaires only are administered. Data collection and meetings are conducted completely remotely, and equipment is either given to them at a doctor’s appointment at the Cancer Center or mailed to and from participants’ homes. Participants will be compensated a total of $100 to complete study activities, $25 for each time point.

This study is being offered in both English and Spanish. Thus, the consent form, study packet, and questionnaires are in both English and Spanish. If a Spanish-speaking coordinator is not available for consent or check-ins, we employ a translator from the UMGCCC.

### The control arm

After randomization, participants meet with the dietitian for individualized nutrition counseling. Despite the existence of guidelines nutrition guidelines for cancer survivors [42], individualized nutrition counseling is often necessary to achieve adherence [43, 44]. Meetings with the dietitian last approximately 30–60 min and focus on key concepts of behavior change including cognitive restructuring and stimulus control [45]. Participants discuss eating habits and set goals based on their individual needs—macronutrients, food groups, minimum servings of nutrient-rich selections, maximum servings of nutrient-poor selections, food preferences, culinary self-efficacy, etc. Participants are not asked to change the timing of their food intake. The participants will meet with the dietitian 7 times throughout the 12-week study (frequency of every two weeks).

### Time-restricted eating (TRE) intervention

Those randomized to TRE self-select a 10-hour eating window based on their schedule and preferences. We encourage the eating window to be during the daytime hours and end approximately 3 h before bedtime. This window should be consistent for the 12-week study duration. Aside from water, which is always allowed, only unsweetened tea and black coffee are allowed before the eating window. Calorie-free foods and beverages such as chewing gum, diet soda, and herbal tea, are discouraged outside the eating window. Participants in the TRE group meet with the same dietitian as those in the control group for individualized nutrition counseling, and goals discussed during the counseling session are asked to be met within the eating window. To match expectancy [46], the two interventions are presented as having a potentially equal benefit on their fatigue (“We are testing two nutritional interventions, both of which should help with your fatigue, one of them has a time component and the other does not”). Those in the TRE group will meet with the same dietitian 7 times throughout the 12-week study, matching the frequency of those in the control group.

### Outcomes

#### Adherence

Eating windows are assessed using the myCircadianClock smartphone application [41] or paper-based logs if requested. This app uses a camera to capture meals and saves entered food items to facilitate data entry. The app has additional features to log sleep, exercise, and medications at the participants’ discretion. This app is user-friendly and has been used in several circadian rhythm clinical trials [11, 17] including cancer populations (e.g., NCT05083416, NCT04783467 [34]). The length of the eating window will be extracted for each participant for each day. Some of our participants have medication requirements that are outside the eating window and recommend concurrent food consumption; if only a small amount of food is ingested (< ~ 25 kcal), this is not included in the eating window. The following parameters will be calculated: (1) how many days the participants ate within a 10-h window, and (2) the average length of the eating window. A participant will be considered “adherent” if they ate within the 10-h window at least 6/7 days (at least 80% of days, measured weekly). (While some TRE protocols build in one or two “cheat” days [33, 47], this is not included as part of our teaching. Also, other protocols use a “95% eating window,” in which 95% of all calorie-containing ingestion events occur [11]. However, we are not confident that we will obtain accurate calorie information at each eating instance to calculate a 95% eating window.) Adherence information will be used in secondary analyses of the effects of TRE on fatigue and other outcomes.

It is likely that there will be some non-adherence in the TRE group, and possible that contamination will exist in the control group, i.e., controls following TRE. Therefore, a secondary per-protocol analysis will be performed in addition to an intent-to-treat analysis.

#### Fatigue

Patient-reported fatigue is the primary outcome, as measured using the Multidimensional Fatigue Symptom Inventory-Short Form (MFSI) [39] which captures five dimensions—general, physical, emotional, and mental fatigue, and vigor. The 30-item fatigue subscale has been validated among patients with cancer [48] and has a published minimal clinically important difference (MCID) of 4.5 points [49]. As an exploratory measure, the BFI [50] is also being administered, which captures fatigue in the last 24 h and how fatigue has interfered with activities. Questionnaires will be administered at baseline, week 6, week 12, and week 24.

#### Circadian rest-activity rhythm via activity tracker

Using a research-grade MotionWatch8, activity is assessed in three axes, recorded as “counts.” The following measures will be extracted from these counts (MotionWare, CamNTech): parametric measures (cosine peak, amplitude, midline estimating statistic of rhythm [MESOR]) and non-parametric measures of activity (interdaily stability, intradaily variability, activity in the 5 consecutive hours with the least activity, activity in the 10 consecutive hours with the most activity, relative amplitude) [34, 51]. By combining these measures with questionnaire data, it can be inferred whether any improvements in fatigue were associated with improved sleep or increased daytime activity. Participants will be asked to wear the watch for at least seven consecutive days at baseline, week 6, and week 12.

#### Continuous glucose monitoring

Interstitial glucose concentrations will be assessed using a FreeStyle Libre continuous glucose monitor (Abbott Labs). The following parameters will be calculated: fasting glucose, average daily glucose, average waking and nocturnal glucose, maximum glucose, area under the curve (AUC) [52], and concordance of glucose curves day-to-day (i.e., average standard deviation across the 7 days). This device is placed on the back of the upper arm for up to two weeks. It is painless, unobtrusive, and does not interfere with bathing, exercising, or swimming. Data are saved automatically every 15 min. Interstitial glucose reflects blood glucose concentrations, though measures are delayed by 4–15 min [53]. The benefit of continuous glucose monitoring over blood glucose is that it avoids frequent, painful finger pricks or venipunctures. Interstitial glucose concentrations will be assessed at baseline, week 6, and week 12.

#### Body weight

Unintentional weight loss has been identified as the largest risk of TRE among cancer survivors by our IRB. More than two-thirds of blood cancer survivors are overweight or obese [54, 55] and, therefore, slow loss of fat mass is a desirable outcome for many patients. However, unintentional weight loss can be a sign of malnutrition [56]. Therefore, participants are asked to measure their body weight weekly. A bathroom scale will be provided and participants will be instructed to place it on a flat, hard surface. Body weight measurements should occur in the morning, shortly after waking, to obtain the most reliable measurements. Study staff will ask about body weight changes at the biweekly check ins, and ask participants to alert the team if body weight drops more than 3% in one week or 5% body weight every two weeks, consistent with the Common Terminology Criteria for Adverse Events (CTCAE), version 5 [57].

#### Other variables

All participants will complete baseline demographic variables and measures of mood (Positive and Negative Affect Schedule [PANAS]-Short Form) [58]; sleep (Insomnia Sleep Index [ISI]) [59]; physical activity (Global Physical Activity Questionnaire [GPAQ]); as well as depression, anxiety, pain, and cognitive problems (Symptom Inventory with a 11-point Likert scale [60] including how much symptoms interfere with daily activities) at baseline, week 6, week 12, and week 24. We will assess potential mediating effects with measures over time because they commonly co-occur with fatigue [61]. Specifically, both *total effects* of the intervention (effect of TRE + changes in clustering symptoms) and the *direct effects* of the intervention can be examined by isolating effects from TRE.

### Data management

#### Confidentiality

Only the principal investigator and her trained and designated research personnel will have access to confidential information. All confidential information that includes personally identifiable information will be coded with a study identification number. The principal investigator and study coordinator(s) will be the only individuals with access to the key of the assigned identification numbers. All confidential information will be locked in a cabinet in a secured location at the University of Maryland School of Nursing or in a database hosted on University of Maryland Baltimore-hosted HIPAA-compliant servers accessible only with a personal password. Personally identifiable information will not be used for this study’s analyses. Everyone using study information will work to keep personal information confidential, and personal information will not be given out unless required by law.

#### Data organization and cleaning

Data will be entered into REDCap electronic forms and electronically collated in a HIPAA-compliant REDCap database managed by University of Maryland Baltimore. Data collected from the myCircadianClock app will be checked in to the Salk Institute. After collation, data will be audited electronically and visually, as needed, for errors.

Whenever possible, cleaning of data from questionnaires, actigraphs, glucose monitors, and other variables will be performed by a blinded researcher. Appropriate graphical or statistical diagnostics methods will be employed in each of the analyses to evaluate distribution of variables (e.g., to identify shape of the distribution and outliers) and also to evaluate the model assumptions (e.g., if deviation from the statistical assumptions is found, we will use alternative methods such as transformations [62] or nonparametric analyses [63, 64]). Outliers will be investigated to determine if they are due to error. If not, analyses will be conducted with and without them to assess sensitivity. Unless stated otherwise, all hypothesis testing will be at the two-sided 0.05 level for type I error as appropriate for phase II trials. SAS, Mplus, and/or JMP will be used for the analyses. Following the intent-to-treat principle, all randomized survivors will be analyzed in the arm as allocated, regardless of their compliance with the intervention or restricted eating contamination in the control group.

In regard to missing data, every effort will be made to facilitate participants’ completion of questionnaires and provision of device data. However, some missing data are inevitable. The reasons for missing data will be recorded and tabulated according to treatment group. If more than 5% missing is observed, missingness mechanism and patterns will be conducted and appropriate approaches employed to minimize the biased estimation. If the missing appears to be *missing at random* [65], we will use multiple imputation or the maximum likelihood method to obtain unbiased estimates of key statistics. If data are suspected to be *missing not at random*, a sensitivity analysis using selection and pattern mixture models will be run to determine the impact on results [66]. If the estimates are similar to the ones obtained from the simpler analysis of only complete cases, we will report the complete-case analysis results [67].

### Statistical analysis

#### Sample size and power calculation

The protocol is approved to recruit 96 participants. They will be randomized in a 1:1 ratio, intervention: control (48:48) using computer-generated block randomization with blocks of 2 or 4. The estimated attrition is approximately 20%, thus the estimated retention rate is 76 (38:38) evaluable participants. This sample size is appropriate for phase II randomized studies and it will allow us to obtain preliminary estimates of descriptive statistics [mean, standard deviation (SD), confidence intervals (CI), and correlations between repeated measures] [68]. The primary endpoint is the change in fatigue as measured by the MFSI [39] from baseline (T1) to 12 weeks (T3), with a midpoint assessment at 6 weeks (T2). The sample size calculation was based on repeated measure analysis of variance (ANOVA). Assuming correlations [69] of 0.60 between repeated MFSI measurements, a total sample size of *n* = 76 will provide 80% power at the 0.05 two-sided significance level to detect a medium effect size (difference in change between groups divided by the baseline SD of the MFSI score) of 0.50 for the intervention effect. The estimate of effect size is based on our feasibility trial and studies with similar interventions. Our feasibility trial [18] yielded an effect size of 0.55 (within-subject, pre- to post-intervention change) using the Functional Assessment of Chronic Illness Therapy-Fatigue (FACIT-F) questionnaire [70], fatigue subscale, which is likely an overestimate of between-group differences. We decided to employ the MFSI instead of the FACIT-F for this study because the MFSI captures multidimensional aspects of fatigue; the FACIT-F captures multidimensional aspects of quality of life including fatigue, therefore the MFSI is a more appropriate construct for a primary outcome of fatigue.

#### Data analysis

Prior to hypothesis testing, the demographics and patients’ characteristics will be compared between TRE vs. control groups and the relevant variables (e.g., age) that differ by group will be included as confounders in hypotheses testing. To provide estimates of efficacy comparing TRE vs. an unrestricted eating pattern to alleviate fatigue, the first objective is to longitudinally evaluate the between-arm difference in mean change in MFSI score from baseline to post-intervention (12 weeks after baseline) in a linear mixed model. A data collection point at 6 weeks is also included in order to capture any effects of the intervention at a shorter time point. The outcome will be MFSI score and the fixed effects will include arm (TRE vs. control), time (baseline, 6, and 12 weeks), and interactions between arm and time. Statistical significance of the arm×time interaction will be assessed at an alpha of 0.05. Participants will be included as a random effect accommodating the correlations between repeated measures. If any aforementioned potential confounders (e.g., age, body mass index, psychological symptoms, time since completion of treatment) are not evenly distributed between the two groups, they will be added to the model. The results of the analyses together with the estimates of distribution parameters (e.g., mean, SD) of MFSI values as well as the change scores will be used in planning future randomized trials. In addition to statistical significance, we will assess the effect sizes and whether any changes seen are clinically meaningful. The MFSI has a published MCID of 4.5 points [49].

A second objective is to assess the sustainability of TRE three months after the intervention. At 24 weeks, follow-up will occur with several questionnaires. Participants will be asked to self-report the time of their typical first food/beverage intake and their typical last food/beverage intake at night over the previous week. The eating window will be calculated, and its length will be compared between arms using a t-test. We will also evaluate the proportion of patients adhering to 10-h window. We hypothesize those in the intervention group will have adopted a significantly shorter eating window than those in the control group.

Mechanistic objectives are to assess intervention effects on rest-activity rhythms and glucose parameters. Similar models as for our primary objective will be constructed with appropriate dependent variables. The statistical results will be adjusted for multiple testing using the Benjamini-Hochberg method [71] at a q = 0.15 level.

### Protocol amendments

All protocol amendments need to be approved by the IRB. Protocol amendments that include eligibility criteria, study procedures, outcomes, payment methods, etc. will be communicated by updating clinicaltrials.gov and/or directly to the relevant parties involved (e.g., active participants), as appropriate.

## Results

This trial opened to recruitment in September 2024. It will likely recruit through 2027 and results of the primary objective are expected to be available in 2028. Results of this trial will be shared on clinicaltrials.gov; in published manuscripts that will be freely accessible at least through PubMed Central; at local, national, and international conferences; and local community groups. Authorship for manuscripts will be determined based on journal requirements but, in general, will require each author to make an intellectual contribution to the project. Raw de-identified data files will be shared using Dryad (datadryad.org) per guidelines of the data repository, or a similar repository, including all de-identified demographic data, clinical record data, and arm allocation from the parent trial. The blank questionnaires and interview guides will also be shared via the same repository, as well as any relevant statistical code.

## Discussion

This trial has many strengths that will increase its impact. First, recruitment is occurring from a Comprehensive Cancer Center affiliated with a university in Baltimore, Maryland, which serves a catchment area where approximately half identify as being from races and ethnic groups who have historically been underrepresented in this scope of research. We are also offering our study to both English- and Spanish-speakers. Thus, we are hopeful that our cohort is generalizable to the vast majority of American cancer survivors. Second, all study activities are conducted completely remotely, facilitating the ability to implement and disseminate a TRE program into many clinical practices in the future. Third, we have an active control condition to help control for time, attention, expectation of benefit, and potential improvements in the *quality* of diet, which may help discern the specific effects of the TRE eating pattern.

However, this study was not without limitations. Our cohort will be heterogeneous in regard to blood cancer types (i.e., leukemia, lymphoma, and multiple myeloma) and treatment history; while that may increase generalizability, it may reduce our ability to see benefits if TRE is only effective for a subset of the eligible participants. Second, we are relying on participants to self-report eating windows and will not be able to confirm their accuracy. To encourage accurate reporting, we assure participants that we are not judging them if they do not adhere and will provide encouragement in future weeks. Also, fatigue is a subjective measure by nature and can fluctuate over the course of the day and week. We carefully selected questionnaires to capture fatigue across different time scales to try to most accurately quantify it, though we are also conducting a qualitative interview at the end of the study to capture any lived experience that cannot be captured via questionnaires. Lastly, we do not know how long a TRE study needs to be implemented to achieve clinically meaningful effects [72]. Further research will be necessary to determine how long an individual will need to adhere to TRE to benefit.

This work will have immediate clinical implications. Because TRE with a 10-hour eating window is generally safe, upon completion of this study, oncology dietitians and nurses seeing people living with cancer as a chronic condition will have evidence for or against inclusion of nutrient timing in their nutrition teaching.

This work also has immediate research implications, and data generated herein will be leveraged in at least three ways. First, it will be used to optimize TRE as an intermittent fasting program. These data will be integrated with data from other studies that have implemented other TRE protocols to compare adherence and efficacy. For example, some programs employ an 8-hour eating window (e.g., [73]), some allow the window to change every day based on a person’s schedule (e.g., [47]), some allow “cheat” days (e.g., [33]), and some dictate whether the window should be “earlier” or “later” in the day (e.g., [74]). Others are combining TRE with exercise (e.g., [75]) or a specific diet (e.g., Mediterranean Diet [76]). These data will be integrated to help clinicians, patients, and caregivers select the TRE protocol that will be the most sustainable and effective for them and their goals. Second, these data will be leveraged in a larger, phase III multisite clinical trial, for example in the NCI Community Oncology Research Program (NCORP) network, to accelerate incorporation of nutrient timing into nutrition guidelines for cancer survivors. Third, these data will help elucidate the etiology of cancer-related fatigue to allow for the development of predictive biomarkers, diagnostics, and complete treatments for fatigue. Specifically, future work will explore molecular changes over the course of the intervention using biological samples (e.g., blood, saliva, urine) to further optimize the intervention. Follow-on studies will continue to test composition, timing, and quantity of nutrients to improve the specificity of nutrition guidelines for cancer survivors. Future studies will look specifically at people who work “second shift,” “third shift,” or other overnight positions, who comprise 16% of the US workforce [77]; recent research demonstrates that eating during the light period improves mood despite working overnight shifts [78]. Based on the findings herein and the growing body of literature on TRE, TRE has potential as a therapeutic for cancer-related fatigue in survivors of other types of cancer, especially ones that have a high 5-year survival rate and high levels of persistent fatigue such as breast cancer and gastrointestinal cancers.

In conclusion, this phase II RCT will examine the effects of TRE on cancer-related fatigue as a primary objective and examine mechanisms by which eating patterns, circadian rhythms, and cancer-related fatigue are associated. Data generated herein will inform clinical practice and future research into how to exploit circadian processes to alleviate cancer-related fatigue and other issues of supportive care in cancer.

## Supplementary Information

Supplementary Material 1.

Supplementary Material 2.

## Data Availability

No datasets were generated or analysed during the current study.

## Abbreviations

CAR: Chimeric antigen receptor
DSM/QAC: Data and Safety Monitoring/Quality Assurance Committee
HIPAA: Health Insurance Portability and Accountability Act
HPA: Hypothalamic–pituitary–adrenal
RCT: Randomized controlled trial
TRE: Time-restricted eating
UMGCCC: University of Maryland Marlene and Stewert Greenebaum Comprehensive Cancer Center

## References

[1] Al Maqbali M, Al Sinani M, Al Naamani Z, Al Badi K, Tanash MI. Prevalence of fatigue in patients with cancer: a systematic review and meta-analysis. J Pain Symptom Manage. 2020;61:167–89. 32768552 10.1016/j.jpainsymman.2020.07.037

[2] Ma Y, He B, Jiang M, Yang Y, Wang C, Huang C, et al. Prevalence and risk factors of cancer-related fatigue: a systematic review and meta-analysis. Int J Nurs Stud. 2020;111:103707. 32920423 10.1016/j.ijnurstu.2020.103707

[3] Bower JE. The role of neuro-immune interactions in cancer-related fatigue: biobehavioral risk factors and mechanisms. Cancer. 2019;125:353–64. 30602059 10.1002/cncr.31790 PMC6502236

[4] Berger AM, Mooney K, Alvarez-Perez A, Breitbart WS, Carpenter KM, Cella D, et al. Cancer-related fatigue, version 2.2015, clinical practice guidelines in Oncology. Nat Comp Cancer Netw. 2015;13(8):1012–39. 10.6004/jnccn.2015.0122 PMC5499710 26285247

[5] Saligan LN, Olson K, Filler K, Larkin D, Cramp F, Yennurajalingam S, et al. The biology of cancer-related fatigue: a review of the literature. Support Care Cancer. 2015;23:2461–78. 25975676 10.1007/s00520-015-2763-0 PMC4484308

[6] Yang S, Chu S, Gao Y, Ai Q, Liu Y, Li X, et al. A narrative review of Cancer-related fatigue (CRF) and its possible pathogenesis. Cells. 2019. 10.3390/cells8070738. 31323874 10.3390/cells8070738 PMC6679212

[7] Abbott SM, Malkani RG, Zee PC. Circadian disruption and human health: A bidirectional relationship. Eur J Neurosci. 2018. 10.1111/ejn.14298 PMC7261021 30549337

[8] Payne JK. Altered circadian rhythms and cancer-related fatigue outcomes. Integr Cancer Ther. 2011;10:221–33. 21382964 10.1177/1534735410392581

[9] Roscoe JA, Morrow GR, Hickok JT, Bushunow P, Matteson S, Rakita D, et al. Temporal interrelationships among fatigue, circadian rhythm and depression in breast cancer patients undergoing chemotherapy treatment. Support Care Cancer. 2002;10:329–36. 12029433 10.1007/s00520-001-0317-0

[10] Forbes-Robertson S, Dudley E, Vadgama P, Cook C, Drawer S, Kilduff L. Circadian disruption and remedial interventions. Sports Med. 2012;42:185–208. 22299812 10.2165/11596850-000000000-00000

[11] Wilkinson MJ, Manoogian ENC, Zadourian A, Lo H, Fakhouri S, Shoghi A, et al. Ten-Hour Time-Restricted eating reduces Weight, blood Pressure, and atherogenic lipids in patients with metabolic syndrome. Cell Metab. 2020;31:1–13. 31813824 10.1016/j.cmet.2019.11.004 PMC6953486

[12] Sullivan KA, Grant CV, Jordan KR, Obrietan K, Pyter LM. Paclitaxel chemotherapy disrupts behavioral and molecular circadian clocks in mice. Brain Behav Immun. 2022;99:106–18. 34563619 10.1016/j.bbi.2021.09.011 PMC8671246

[13] Sultan A, Choudhary V, Parganiha A. Worsening of rest-activity circadian rhythm and quality of life in female breast cancer patients along progression of chemotherapy cycles. Chronobiol Int. 2017;34:609–23. 28276853 10.1080/07420528.2017.1286501

[14] Li W, Kwok CC, Chan DC, Ho AW, Ho CS, Zhang J, et al. Disruption of sleep, sleep-wake activity rhythm, and nocturnal melatonin production in breast cancer patients undergoing adjuvant chemotherapy: prospective cohort study. Sleep Med. 2019;55:14–21. 30743205 10.1016/j.sleep.2018.11.022

[15] Ancoli-Israel S, Liu L, Natarajan L, Rissling M, Neikrug AB, Youngstedt SD, et al. Reductions in sleep quality and circadian activity rhythmicity predict longitudinal changes in objective and subjective cognitive functioning in women treated for breast cancer. Support Care Cancer. 2022;30:3187–200. 34957532 10.1007/s00520-021-06743-3 PMC8857013

[16] Zarrinpar A, Chaix A, Panda S. Daily eating patterns and their impact on health and disease. Trends Endocrinol Metab. 2016;27:69–83. 26706567 10.1016/j.tem.2015.11.007 PMC5081399

[17] Gill S, Panda S. A smartphone app reveals erratic diurnal eating patterns in humans that can be modulated for health benefits. Cell Metab. 2015;22:789–98. 26411343 10.1016/j.cmet.2015.09.005 PMC4635036

[18] Kleckner AS, Altman BJ, Reschke JE, Kleckner IR, Culakova E, Dunne RF, et al. Time-restricted eating to address cancer-related fatigue among cancer survivors: A single-arm pilot study. J Integr Oncol. 2022;11:1–6. PMC9489052 36131848

[19] Chaix A, Manoogian ENC, Melkani GC, Panda S. Time-restricted eating to prevent and manage chronic metabolic diseases. Annu Rev Nutr. 2019;39:291–315. 31180809 10.1146/annurev-nutr-082018-124320 PMC6703924

[20] Melkani GC, Panda S. Time-restricted feeding for prevention and treatment of cardiometabolic disorders. J Physiol. 2017;595:3691–700. 28295377 10.1113/JP273094 PMC5471414

[21] Christensen RAG, Kirkham AA, Time-Restricted Eating. A novel and simple dietary intervention for primary and secondary prevention of breast cancer and cardiovascular disease. Nutrients. 2021;13(10):3476. 10.3390/nu13103476. 10.3390/nu13103476 PMC8537890 34684476

[22] Hatori M, Vollmers C, Zarrinpar A, DiTacchio L, Bushong EA, Gill S, et al. Time-restricted feeding without reducing caloric intake prevents metabolic diseases in mice fed a high-fat diet. Cell Metab. 2012;15:848–60. 22608008 10.1016/j.cmet.2012.04.019 PMC3491655

[23] Gabel K, Hoddy KK, Haggerty N, Song J, Kroeger CM, Trepanowski JF, et al. Effects of 8-hour time restricted feeding on body weight and metabolic disease risk factors in obese adults: a pilot study. Nutr Health Aging. 2018;4:345–53. 10.3233/NHA-170036 PMC6004924 29951594

[24] Jamshed H, Beyl RA, Della Manna DL, Yang ES, Ravussin E, Peterson CM. Early time-restricted feeding improves 24-hour glucose levels and affects markers of the circadian clock, aging, and autophagy in humans. Nutrients. 2019. 10.3390/nu11061234. 31151228 10.3390/nu11061234 PMC6627766

[25] Wang XS. Clinical factors associated with cancer-related fatigue in patients being treated for leukemia and Non-Hodgkin’s lymphoma. J Clin Oncol. 2002;20:1319–28. 11870175 10.1200/JCO.2002.20.5.1319

[26] Suzuki K, Kobayashi N, Ogasawara Y, Shimada T, Yahagi Y, Sugiyama K, et al. Clinical significance of cancer-related fatigue in multiple myeloma patients. Int J Hematol. 2018;108:580–7. 30155589 10.1007/s12185-018-2516-1

[27] 2025 31 January 2025. Cancer Stat Facts: Leukemia. National Cancer Institute, <. https://seer.cancer.gov/statfacts/html/leuks.html. 31 January 2025.

[28] 2025 31 January 2025. Cancer Stat Facts: Myeloma. National Cancer Institute, <. https://seer.cancer.gov/statfacts/html/mulmy.html. 31 January 2025.

[29] National Cancer Institute. 2025 31 January 2025. Cancer Stat Facts: Non-Hodgkin Lymphoma. https://seer.cancer.gov/statfacts/html/nhl.html. 31 January 2025.

[30] 2025 Cancer Stat Facts: Hodgkin Lymphoma. National Cancer Institute, <. https://seer.cancer.gov/statfacts/html/hodg.html. Accessed 31 January 2025.

[31] O’Donnell E, Shapiro Y, Comander A, Isakoff S, Moy B, Spring L, et al. Pilot study to assess prolonged overnight fasting in breast cancer survivors (longfast). Breast Cancer Res Treat. 2022;193:579–87. 35441995 10.1007/s10549-022-06594-4

[32] Kirkham AA, Ford KL, Ramos Da Silva B, Topolnyski J, Prado CM, Joy AA, et al. Implementation of weekday time-restricted eating to improve metabolic health in breast cancer survivors with overweight/obesity. Obes (Silver Spring). 2023;31(Suppl 1):150–60. 10.1002/oby.23654 36695128

[33] Kirkham AA, Ford KL, Topolnyski J, Da Silva BR, Paterson DI, Prado CM, et al. Time-Restricted eating to reduce cardiovascular risk among older breast cancer survivors: A Single-Arm feasibility study. JACC CardioOncol. 2022;4:276–8. 35818550 10.1016/j.jaccao.2022.03.002 PMC9270634

[34] Kleckner AS, Clingan CL, Youngblood SM, Kleckner IR, Quick L, Elrod RD et al. Time-restricted eating to address persistent cancer-related fatigue among cancer survivors: A pilot randomized controlled trial. Support Care Cancer. 2025;33(4). 10.1007/s00520-025-09394-w. 10.1007/s00520-025-09394-w PMC12547982 40186671

[35] Christensen RAG, Haykowsky MJ, Nadler M, Prado CM, Small SD, Rickard JN, et al. Rationale and design of IMPACT-women: a randomised controlled trial of the effect of time-restricted eating, healthy eating and reduced sedentary behaviour on metabolic health during chemotherapy for early-stage breast cancer. Br J Nutr. 2023;130:852–9. 36453589 10.1017/S0007114522003816 PMC10404477

[36] Figueiredo JC, Salvy S-J, Peterson CM. Time-Restricted eating and cancer: clinical outcomes, mechanisms, and moderators NCT04722341. clinicaltrials.gov. National Cancer Institute. 2023. https://clinicaltrials.gov/study/NCT04722341?term=NCT04722341.

[37] Harris PA, Taylor R, Minor BL, Elliott V, Fernandez M, O’Neal L, et al. The REDCap consortium: Building an international community of software platform partners. J Biomed Inf. 2019;95:103208. 10.1016/j.jbi.2019.103208 PMC7254481 31078660

[38] Lawrence CE, Dunkel L, McEver M, Israel T, Taylor R, Chiriboga G, et al. A REDCap-based model for electronic consent (eConsent): moving toward a more personalized consent. J Clin Transl Sci. 2020;4:345–53. 33244416 10.1017/cts.2020.30 PMC7681162

[39] Stein KD, Martin SC, Hann DM, Jacobsen PB. A multidimensional measure of fatigue for use with cancer patients. Cancer Pract. 1998;6:143–52. 9652245 10.1046/j.1523-5394.1998.006003143.x

[40] Godin G. A simple method to assess exercise behavior in the community. Can J Appl Sport Sci. 1985;10:141–6. 4053261

[41] Manoogian ENC, Wei-Shatzel J, Panda S. Assessing temporal eating pattern in free living humans through the mycircadianclock app. Int J Obes (Lond). 2022. 10.1038/s41366-021-01038-3. 34997205 10.1038/s41366-021-01038-3 PMC9678076

[42] Rock CL, Thomson CA, Sullivan KR, Howe CL, Kushi LH, Caan BJ, et al. American Cancer Society nutrition and physical activity guideline for cancer survivors. CA Cancer J Clin. 2022;72(3):230–62. 35294043 10.3322/caac.21719

[43] James S, Oppermann A, Schotz K, Minotti M, Rao GG, Baguley BJ et al. Dietary interventions during chemotherapy treatment: a systematic review of safety, feasibility, and efficacy. Curr Oncol. 2025;32(1):3. 10.3390/curroncol32010003. 10.3390/curroncol32010003 PMC11764068 39851919

[44] Oppermann A, James S, Minotti M, Schotz K, Francis ME, Kleckner IR, et al. Dietary interventions during radiation therapy: A systematic review of feasibility, safety, and efficacy. Nutr Cancer. 2025;77:26–50. 39340400 10.1080/01635581.2024.2406999 PMC12616625

[45] Spahn JM, Reeves RS, Keim KS, Laquatra I, Kellogg M, Jortberg B, et al. State of the evidence regarding behavior change theories and strategies in nutrition counseling to facilitate health and food behavior change. J Am Diet Assoc. 2010;110:879–91. 20497777 10.1016/j.jada.2010.03.021

[46] Boot WR, Simons DJ, Stothart C, Stutts C. The pervasive problem with placebos in psychology: why active control groups are not sufficient to rule out placebo effects. Perspect Psychol Sci. 2013;8:445–54. 26173122 10.1177/1745691613491271

[47] James DL, Larkey LK, Maxfield M, Han S, Ofori E, Mohr AE, et al. Prolonged nightly fasting in older adults with memory decline: a single-group pilot study exploring changes in cognitive function and cardiometabolic risk factors. J Clin Transl Sci. 2025;9(1):e1. 39830610 10.1017/cts.2024.676 PMC11736298

[48] Stein KD, Jacobsen PB, Blanchard CM, Thors C. Further validation of the multidimensional fatigue symptom inventory-short form. J Pain Symptom Manage. 2004;27:14–23. 14711465 10.1016/j.jpainsymman.2003.06.003 PMC2547485

[49] Chan A, Yo TE, Wang XJ, Ng T, Chae JW, Yeo HL, et al. Minimal clinically important difference of the multidimensional fatigue symptom Inventory-Short form (MFSI-SF) for fatigue worsening in Asian breast cancer patients. J Pain Symptom Manage. 2018;55:992–7. e2. 29097274 10.1016/j.jpainsymman.2017.10.014

[50] Mendoza TR, Wang XS, Cleeland CS, Morrissey M, Johnson BA, Wendt JK, et al. The rapid assessment of fatigue severity in cancer patients: use of the brief fatigue inventory. Cancer. 1999;85:1186–96. 10091805 10.1002/(sici)1097-0142(19990301)85:5<1186::aid-cncr24>3.0.co;2-n

[51] Rogers VE, Zhu S, Mandrell BN, Ancoli-Israel S, Liu L, Hinds PS. Relationship between circadian activity rhythms and fatigue in hospitalized children with CNS cancers receiving high-dose chemotherapy. Support Care Cancer. 2020;28:1459–67. 31273507 10.1007/s00520-019-04960-5 PMC7426544

[52] Hutchison AT, Regmi P, Manoogian ENC, Fleischer JG, Wittert GA, Panda S, et al. Time-restricted feeding improves glucose tolerance in men at risk for type 2 diabetes: a randomized crossover trial. Obesity. 2019;27:724–32. 31002478 10.1002/oby.22449

[53] Dye L, Mansfield M, Lasikiewicz N, Mahawish L, Schnell R, Talbot D, et al. Correspondence of continuous interstitial glucose measurement against arterialised and capillary glucose following an oral glucose tolerance test in healthy volunteers. Br J Nutr. 2010;103:134–40. 19674490 10.1017/S0007114509991504

[54] Lynce F, Pehlivanova M, Catlett J, Malkovska V. Obesity in adult lymphoma survivors. Leuk Lymphoma. 2012;53:569–74. 21888618 10.3109/10428194.2011.619606

[55] Vogl DT, Wang T, Perez WS, Stadtmauer EA, Heitjan DF, Lazarus HM, et al. Effect of obesity on outcomes after autologous hematopoietic stem cell transplantation for multiple myeloma. Biol Blood Marrow Transplant. 2011;17:1765–74. 21624486 10.1016/j.bbmt.2011.05.005 PMC3175301

[56] Kleckner AS, Magnuson A. The nutritional needs of older cancer survivors. J Geriatr Oncol. 2022;13:738–41. 34906443 10.1016/j.jgo.2021.12.007 PMC9187777

[57] U.S. Department of Health and Human Services. Common Terminology Criteria for Adverse Events (CTCAE), version 5.0. 2017.

[58] Thompson ER. Development and validation of an internationally reliable short-form of the positive and negative affect schedule (PANAS). Journal of Cross-Cultural Psychology. 2007;38:227–42.

[59] Bastien CH, Vallieres A, Morin CM. Validation of the insomnia severity index as an outcome measure for insomnia research. Sleep Med. 2001;2:297–307. 11438246 10.1016/s1389-9457(00)00065-4

[60] Cleeland C, Mendoza TR, Wang XS, Chou C, Harle MT, Morrissey M, et al. Assessing symptom distress in cancer patients: the M. D. Anderson symptom inventory. Cancer. 2000;89:1634–46. 11013380 10.1002/1097-0142(20001001)89:7<1634::aid-cncr29>3.0.co;2-v

[61] George MA, Lustberg MB, Orchard TS. Psychoneurological symptom cluster in breast cancer: the role of inflammation and diet. Breast Cancer Res Treat. 2020;184:1–9. 32803635 10.1007/s10549-020-05808-x

[62] Box GEP, Cox DR. An analysis of transformations revisited, rebutted. J Am Stat Assoc. 1982;77:209–10.

[63] Conover W, Iman R. On some alternative procedures using ranks for the analysis of experimental designs. Commun Stat. 1976;5:1349–68.

[64] Conover W, Iman R. Rank transformations as a bridge between parametric and nonparametric statistics. Am Stat. 1981;35:124–33.

[65] Carpenter JR, Kenward MG. 2007 Missing data in randomised controlled trials - a practical guide. http://www.pcpoh.bham.ac.uk/publichealth/nccrm/PDFs_and_documents/Publications/Final_Report_RM04_JH17_mk.pdf.

[66] Little RJA. Pattern-mixture models for multivariate incomplete data. J Am Stat Assoc. 1993;88:125–34.

[67] Molenberghs G, Kenward MG. Missing data in clinical studies. West Sussex: John Wiley and Sons; 2007.

[68] Hertzog MA. Considerations in determining sample size for pilot studies. Res Nurs Health. 2008;31:180–91. 18183564 10.1002/nur.20247

[69] Borm GF, Fransen J, Lemmens WA. A simple sample size formula for analysis of covariance in randomized clinical trials. J Clin Epidemiol. 2007;60:1234–8. 17998077 10.1016/j.jclinepi.2007.02.006

[70] Cella D. The functional assessment of cancer therapy-anemia (FACT-An) scale: a new tool for the assessment of outcomes in cancer anemia and fatigue. Semin Hematol. 1997;34:13–9. 9253779

[71] Benjamini Y, Hochberg Y. Controlling the false discovery rate - a practical and powerful approach to multiple testing. Journal of the Royal Statistical Society Series B: Statistical Methodology. 1995;57:289–300.

[72] Kirkham AA, Parr EB, Kleckner AS. Cardiometabolic health impacts of time-restricted eating: implications for type 2 diabetes, cancer, and cardiovascular diseases. Curr Opin Clin Nutr Metab Care. 2022;25:378–87. 36017558 10.1097/MCO.0000000000000867 PMC9990131

[73] Wingo BC, Rinker JR 2nd, Green K, Peterson CM. Feasibility and acceptability of time-restricted eating in a group of adults with multiple sclerosis. Front Neurol. 2022;13:1087126. 36712417 10.3389/fneur.2022.1087126 PMC9878382

[74] Jamshed H, Steger FL, Bryan DR, Richman JS, Warriner AH, Hanick CJ, et al. Effectiveness of early time-restricted eating for weight loss, fat loss, and cardiometabolic health in adults with obesity: a randomized clinical trial. JAMA Intern Med. 2022;182:953–62. 35939311 10.1001/jamainternmed.2022.3050 PMC9361187

[75] Miladi S, Driss T, Ameur R, Miladi SC, Miladi SJ, Najjar MF, et al. Effectiveness of early versus late time-restricted eating combined with physical activity in overweight or obese women. Nutrients. 2025. 10.3390/nu17010169. 39796603 10.3390/nu17010169 PMC11723088

[76] Karras SN, Koufakis T, Popovic DS, Adamidou L, Karalazou P, Thisiadou K, et al. A mediterranean eating pattern combining energy and time-restricted eating improves Vaspin and omentin concentrations compared to intermittent fasting in overweight individuals. Nutrients. 2023. 10.3390/nu15245058. 38140318 10.3390/nu15245058 PMC10745393

[77] US Bureau of Labor Statistics. Job flexibilities and work schedules summary. 2019. https://www.bls.gov/news.release/flex2.nr0.htm.

[78] Qian J, Vujovic N, Nguyen H, Rahman N, Heng SW, Amira S, et al. Daytime eating prevents mood vulnerability in night work. Proc Natl Acad Sci U S A. 2022;119:e2206348119. 36095195 10.1073/pnas.2206348119 PMC9499546

