# Supplementary material for: Take the reins: a study protocol of a randomized controlled trial testing the effects of time-restricted eating vs. nutrition control on cancer-related fatigue among survivors of hematological malignancies
Source: BMC Nutr. 2025 Oct 31;11:200. doi: 10.1186/s40795-025-01185-0 (PMC12577101; doi:10.1186/s40795-025-01185-0)
Supplement: Supplementary file 1 — Supplementary Material 1. [file 40795_2025_1185_MOESM1_ESM.pdf]

RESEARCH CONSENT FORM AND HIPAA AUTHORIZATION

Protocol Title: Take the Reins: The effects of nutrient timing on cancer-related fatigue among blood cancer survivors

Study Number: HP-00110284

Principal Investigator: Amber Kleckner, PhD, 410-706-5961

Sponsor: National Institutes of Health (NIH) National Cancer Institute (NCI)

This Consent document describes a research study, what you may expect if you decide to take part, and important information to help you make your decision. Please read this form carefully and ask questions before you agree to participate.

CONCISE SUMMARY:

Most patients experience tiredness during cancer treatment, referred to as "cancer-related fatigue." For many people, their tiredness does not go away after treatment. Doctors and scientists do not fully understand why the fatigue occurs, and we do not have good ways to treat it. There are new data suggesting that fatigue could arise from a person's body clock (i.e., circadian rhythm) being out of alignment. However, this has not been proven. In this study, we will collect data on daily activities that are related to biological rhythms—sleep patterns, physical activity, and diet—to get more information on the relationships between circadian rhythm and fatigue.

The study lasts approximately 14 weeks, with a follow-up at 24 weeks. At the beginning of the study, you will be asked to complete online questionnaires related to your symptoms and feelings. For one week, we will ask you to log your diet and sleep using a smartphone app called myCircadianClock. For the same week, we will ask you to wear an activity tracker on your wrist (like a watch) as well as a continuous glucose monitor on the back of your upper arm. After the first week, everyone will meet with a nutritionist to discuss nutrition in cancer survivorship. You will be randomly assigned to one of two groups: one group will be asked to eat all their food within a 10-hour window during the day for 12 weeks (you pick the start time, for example 9am-7pm); the other group will not be asked to change *when* they are eating. At the middle (week 6) and end (week 12) of the study, we will ask you again to complete the same study activities as at the beginning (questionnaires, log diet and sleep, wear the activity tracker and continuous glucose monitor). After 24 weeks, we will ask you to complete an additional set of questionnaires. Study materials may be provided in-person or via mail—your choice (i.e., you do not have to come in). You will be paid a total of $100 for your time to complete the study activities.

Key risks: discomfort from the activity tracker or glucose monitor; emotional distress; breach of confidentiality; weight loss, gastrointestinal upset, or hunger from time-restricted eating

Participating in this research study is voluntary. Your decision to participate will not affect your healthcare or treatment for your cancer in any way.

PURPOSE OF STUDY

In this study, we want to get more information on the relationships between lifestyle behaviors—sleep patterns, physical activity, and diet—and fatigue. “Time-restricted eating” is when you eat all your food in a well-defined window during the day. We will test whether time-restricted eating (a 10-hour eating window), as compared to a longer eating window, changes a person’s level of fatigue.

Approximately 100 participants will be recruited to take part in this study from the University of Maryland Medical System.

PROCEDURES

Here are the study activities and timeline. Each of the activities is described in more detail below.

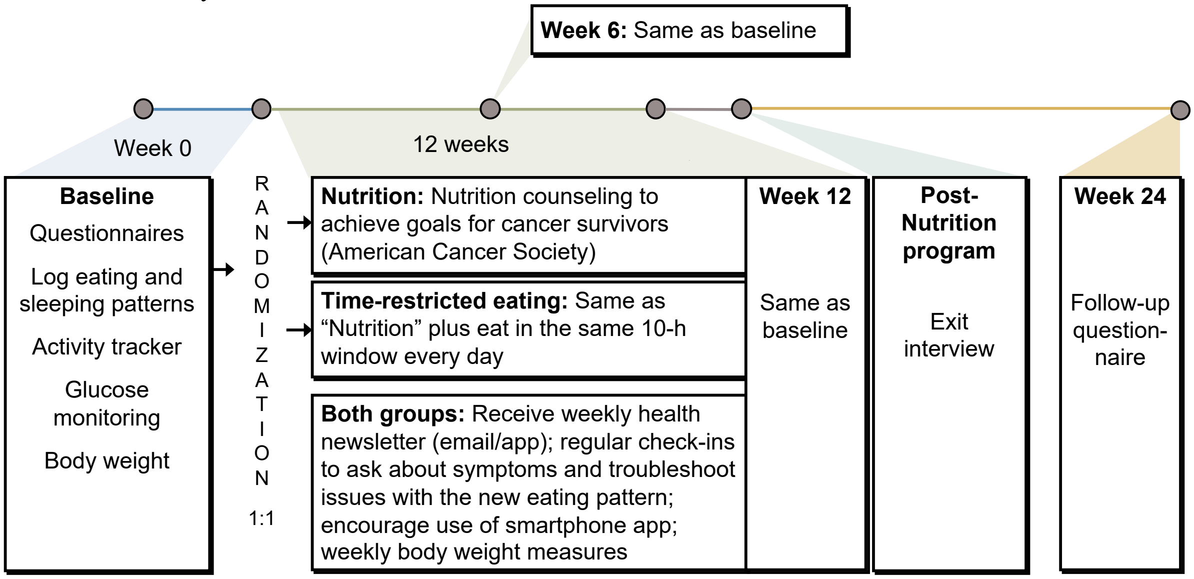

<u>Questionnaires:</u> We will ask you to complete several online questionnaires that ask about your symptoms, feelings, and habits. These will take approximately 30-60 minutes to complete. You also have the choice to complete these on paper. We will ask you to complete questionnaires four times throughout the study—at weeks 0, 6, 12, and 24.

<u>Logging eating and sleeping patterns:</u> We will ask you to log everything you eat using the myCircadianClock app every day at week 0, week 6, and week 12. In addition, we will ask you to log when you go to bed and when you get up using the app. Logging eating and sleeping patterns is optional weeks 1-5 and 7-11. This activity will take approximately 5 minutes per day. You also have the option of recording your food intake on paper.

<u>Actigraphy:</u> We will ask you to wear an activity tracker on your wrist every day for weeks 0, 6, and 12. The activity tracker monitors physical activity.

<u>Glucose monitoring:</u> We will ask you to wear a continuous glucose monitor every day at weeks 0, 6, and 12. This is measuring *interstitial glucose*, which is the concentration of sugar in between your cells and is highly correlated with blood sugar. The continuous glucose monitor is the size of two quarters stacked on

HP-00110284 UM IRB Approval Date 10/1/2024
Do Not Sign this Form after this Date 8/22/2025

top of one another. It is placed on the back of your upper arm. It has a thin, 5-mm-long wire that inserts under the skin and is water-resistant so that it does not bother you. It is applied once at the beginning of the week and you can keep it on for up to 14 days, but we only ask you to wear it for 7 days. We can apply it on-site at the Medical Center, or we can mail it to you to apply yourself. We will instruct you on how to apply the monitor, and we will be available if you have questions when you apply the device to yourself. It feels like a small pinch when you initially put it on.

Body weight: We will provide you with a digital bathroom scale to use at home. Please weigh yourself once per week and enter your weight via the myCircadianClock app or a paper log.

Randomization and the two study groups: After the first week, you will meet with a nutritionist to discuss your diet and nutrition recommendations in cancer survivorship. At this meeting, you will be randomly assigned to one of two groups: the time-restricted eating group or the time-*un*restricted eating group. No one on the study team knows what group you will be in until after week 0. The group you will be in is chosen by chance, like flipping a coin. There is a 50% chance you will be in the time-restricted eating group and 50% chance you will be in the time-unrestricted eating group.

- Time-restricted eating group: If you are assigned to the time-restricted eating group, you will pick a 10-hour window to eat based on your schedule and preferences. For example, this window could start at 7:00am, 9:15am, or another time. Water will be allowed any time. Unsweetened tea and black coffee are allowed in the morning before your eating window. ***All other food and beverages including artificial sweeteners (e.g., chewing gum, diet soda) will be allowed only within the 10-hour eating window.*** Within the 10-hour window, you can eat whatever you want at whatever time, though you will be encouraged to meet the goals you discuss with the nutritionist.
- Time unrestricted eating group: If you are assigned to the time-unrestricted eating group, you will try to meet recommendations discussed with the nutritionist without a restriction on *when* you are eating.

Contact with the study team: We will call you about every two weeks to check in and see how you are doing. You may also call and email the study team in between these check-ins.

myCircadianClock app: In addition to using the app to log eating patterns, sleeping patterns, and body weight, everyone in the study will receive weekly tips on healthy lifestyles through the app.

Exit interview: At the completion of the study, or if you decide to withdraw early, we will “interview” you about your experience in the study. We will ask you what you liked about it, what you didn’t like about it, and ask for feedback on the myCircadianClock app. If you were in the time-restricted eating group, we will ask you about your experiences with the new diet pattern. This interview will take 20-30 minutes and we will record the audio of the conversation (not video) if that is okay with you.

All of these research activities can be done remotely. However, you are welcome to come in and we will help you put on the glucose monitor, use the myCircadianClock app, complete questionnaires, and/or complete the other study activities. We are located in the School of Nursing, across the street from Greenebaum Comprehensive Cancer Center.

HP-00110284 UM IRB Approval Date 10/1/2024
Do Not Sign this Form after this Date 8/22/2025

WHAT ARE MY RESPONSIBILITIES IF I TAKE PART IN THIS RESEARCH STUDY?

If you take part in this study, you will be responsible for participating in the study activities, as outlined above.

POTENTIAL RISKS/DISCOMFORTS:

As with all research studies, there are risks associated with the study activities. We have taken measures to minimize all anticipated risks. Please consider these risks while deciding if you want to participate.

1. *Discomfort from the continuous glucose monitor*
   The continuous glucose monitor is a minimally invasive device that senses sugar just under your skin. It is applied to the back of your upper arm and feels like a pinch when the small, 5-mm-long sensor is inserted. It can also cause pain if the device gets hit while you are wearing it. We selected to use this device rather than obtaining blood glucose measurements because it avoids painful finger pricks.

2. *Emotional distress*
   You could become upset or overwhelmed by the expectation to consume food only in the eating window. However, we want to emphasize that you are not "in trouble" if you do not follow the procedures exactly. We want to use what we learn from this study to improve the procedures for the next study.

   Our questionnaires contain information that might be distressing or private (e.g., "I am satisfied with family communication about my illness"). You do not have to answer any questions you are not comfortable answering, and you can take a break or stop answering the questionnaires at any time.

3. *Breach of confidentiality*
   There is always a risk of a breach of confidentiality in which sensitive medical information could become known to people outside the research team. To avoid leakage of sensitive information, only Dr. Amber Kleckner (the study chair), Carin Clingan (the study coordinator), and any individual designees will have access to the screening log and the file that links your name with your subject number (both will be encrypted); these files will be stored on password-protected computers in their private offices. All data files will reference you by a non-identifiable Participant ID and will be stored on Dr. Kleckner's computer and secure servers at UMB. All consent forms will be stored in a locked cabinet also in her or her staff's office. All audio-recorded interviews will be transferred to Dr. Kleckner's secure computer and server at UMB within 2 business days of the interview and then immediately deleted from the recorder. All interview file names will not include your name or any identifying information. If Dr. Kleckner shares data with any other researcher for analyses, all data will be de-identified (i.e., <u>will not</u> have your name, birthdate, contact information, etc.). Presentation of study findings in the form of presentations and manuscripts, either in private or public settings, will not have any identifiable information, nor will any audio clips ever be played in public. Dr. Kleckner and all other co-investigators participate in ethical training in accordance with institutional policies.

HP-00110284 UM IRB Approval Date 10/1/2024
Do Not Sign this Form after this Date 8/22/2025

4. *Weight loss due to time-restricted eating*
   This is not a weight loss study. Some studies have documented weight loss upon adoption of a new eating pattern despite not "cutting calories" on purpose. We encourage you to eat and drink to satisfy hunger and thirst. However, you will be changing your eating patterns, and there is a chance that you could lose weight during the study. Slow weight loss will not be considered dangerous (less than 3% of body weight per week), especially if you are beginning the study overweight. However, if you experience rapid, unintentional weight loss, as determined by a medical provider, your medical provider will advise you on what to do, which may involve withdrawing from the study.

5. *Gastrointestinal upset*
   With a change in diet, and especially a large change in diet, you could experience constipation, diarrhea, nausea, heartburn, etc. These effects usually go away after several days, but you will always have access to the study team to discuss how to relieve these effects.

6. *Hunger and/or hypoglycemia*
   Those in the time-restricted eating group will be eating their food in a shorter window than normal. If you are assigned to this group, you might get hungry while you are outside your eating window. With hunger can come fatigue, lack of concentration, irritability, headache, and other symptoms. These symptoms will likely go away after a few days, when your body adapts to the new pattern. However, moderate to severe symptoms can be a sign of hypoglycemia (low blood sugar) and we encourage you to eat outside the window if you are feeling sluggish, dizzy, or sick.

7. *Loss of privacy*
   Our study team will access your medical record and talk to you about issues that you might feel are private. You do not need to answer any questions you do not feel comfortable answering.

POTENTIAL BENEFITS

You might or might not benefit from being in this research study. You may become more aware of your diet and sleep patterns, which can contribute to overall health. However, there is no guarantee that you will receive direct benefit from your participation in this study.

ALTERNATIVES TO PARTICIPATION

This is not a treatment study and the alternative is to not participate. Instead of participating, you may choose to log your diet and sleep patterns on your own, and/or see a clinician (e.g., dietitian, primary care physician) to explore how your diet, physical activity, and sleep patterns may be contributing to your fatigue. If you choose not to take part in the study, your decision not to participate should not affect your healthcare.

COSTS TO SUBJECTS

There will be no fee to enroll in the study. You may incur ancillary costs such as travel, lodging, parking, meals, etc.

HP-00110284 UM IRB Approval Date 10/1/2024
Do Not Sign this Form after this Date 8/22/2025

PAYMENT TO SUBJECTS

You will be compensated for your time to complete the study activities. We will pay you a total of $100 to complete all study activities—$25 each for Weeks 0, 6, and 12, which will be paid to you after Week 12 or prorated if you decide to withdraw from the study early. After week 24, you will receive another $25 for completing the questionnaires. These payments will be made in electronic gift cards or via check.

You may need to report payments you receive for participating in the study as taxable income, which could affect your eligibility to receive certain government benefits (e.g., from the Maryland Supplemental Nutrition Assistance Program (SNAP) and the Maryland Temporary Cash Assistance program (TCA)). If you owe a debt to the State of Maryland or the federal government (e.g., child support, taxes), the amount you receive may be reduced.

STUDY-RELATED INJURY

If you have an injury, promptly seek medical care from any healthcare provider. If you have an emergency, call 911 or go to the nearest emergency room. You should tell the healthcare provider that you have participated in a research study.

If you believe the injury is related to the study, notify the study doctor. UMB, if requested, will assist you to get medical care or referrals.

If you are injured as a result of being in this study, you or your insurance will be responsible for paying your medical expenses. Neither the hospital nor UMB has agreed to pay for the cost of medical care or other costs arising from an injury. However, you do not give up any of your legal rights by being in this study, and you may choose to pursue legal action if you are injured by being in the study.

CONFIDENTIALITY AND ACCESS TO RECORDS

Using your medical record number, we will access your electronic medical record so that we can get information regarding your cancer diagnosis, cancer treatment history, medical history, and social history. We will collect your name, address, phone number, and email address in order to contact you for scheduling and reminders of upcoming study activities. Only Dr. Amber Kleckner, the principal investigator, and her trained and designated research personnel will have access to confidential information. All confidential information that includes personally identifiable information will be coded with a study ID number. The principal investigator and study coordinator(s) will be the only individuals with access to the key of the assigned ID numbers. All confidential information will be locked in a cabinet in a secured location at the University of Maryland, School of Nursing. Your personally identifiable information will not be used for this study's analyses, but it will be kept on file if federal agencies or the Institutional Review Board (IRB) are mandated to review any information.

All study records will be considered confidential, and all participants' names and personally identifiable information will not be used in reports or publications. Efforts will be made to limit disclosure of your personal information, including research study and medical records, to people who have a need to review this information. We cannot promise complete secrecy. Organizations that may inspect and copy your information include the IRB and other representatives of the University of Maryland, Baltimore (UMB), or the study site. The monitors, auditors, the IRB, and the National Cancer Institute will be granted direct

access to your medical records for verification of the study procedures and date. By signing this document, you are authorizing this access. Those designated from the University of Maryland will be allowed to examine certain research records of this study; however, anyone inspecting this information is required to keep this personal information confidential. Your personal information will not be released unless mandated by law. By signing this document you are authorizing this access to the monitors, auditors, and the IRB.

The data from the study may be published. However, you will not be identified by name. People designated from the institutions where the study is being conducted, and people from the sponsor will be allowed to inspect sections of your medical and research records related to the study. Everyone using study information will work to keep your personal information confidential. Your personal information will not be given out unless required by law.

A description of this clinical trial is available on http://www.clinicaltrials.gov, as required by U.S. Law. This website will not include information that can identify you. At most, the website will include a summary of the results. You can search this website at any time.

We are using myCircadianClock app to collect data regarding your food intake, sleep patterns, and body weight. The myCircadianClock app was developed by researchers at The Salk Institute in La Jolla, California, and has been used in other research studies. There is detailed information regarding the app at end of this form. **We will not share any personal information (e.g., name) or medical information (e.g., details of your cancer diagnosis) with the myCircadianClock team, though they will have access to any information that you input into the app.** By consenting to this study, you are consenting to have your de-identified app data stored with the Salk Institute and combined with other myCircadianClock data in future analyses. At the end of this study, you may request to have your data removed from their database.

RIGHT TO WITHDRAW

Your participation in this study is voluntary. You do not have to take part in this study. You are free to withdraw your consent at any time. Your refusal to take part or your decision to stop taking part in the study will involve no penalty or loss of benefits to which you are otherwise entitled. If you decide to stop taking part, or if you have questions, concerns, or complaints, or if you need to report a study-related injury, please contact the investigator, Dr. Amber Kleckner, at 410-706-5961. To discontinue your participation in the study, a written withdrawal is requested, sent to Dr. Amber Kleckner at amber.kleckner@umaryland.edu.

If you withdraw from this study, already collected data will not be removed from the study database. You will be asked whether the investigator can collect data from your routine medical care. If you agree, these data will be handled the same as research data.

You will be told of any significant new findings that develop during the study that may affect your willingness to continue participation.

HP-00110284 UM IRB Approval Date 10/1/2024
Do Not Sign this Form after this Date 8/22/2025

CAN I BE REMOVED FROM THE STUDY?

The person in charge of the study or the sponsor can remove you from the study without your approval. Possible reasons for removal include failure to get ahold of you, if your health becomes worse or if your doctor feels that staying in the study is harmful to your health, or if your participation becomes harmful to a study team member. The sponsor can also end the study early. The study team will tell you about this, and you will have the chance to ask questions if this happens.

UNIVERSITY STATEMENT

The University of Maryland, Baltimore (UMB) is committed to providing subjects in its research studies all rights due to them under State and federal law. You give up none of your legal rights by signing this consent form or by participating in this study. This study has been reviewed and approved by an Institutional Review Board (IRB). The IRB is a group of scientists, physicians, experts, and community representatives. The IRB's membership includes persons who are not affiliated with UMB and persons who do not conduct research studies.

If you have questions, concerns, complaints, or believe you have been harmed through participation in this study as a result of researcher negligence, you can contact members of the IRB or the Human Research Protections Office (HRPO) to ask questions, discuss problems or concerns, obtain information, or offer input about your rights as a research study subject. The contact information for the IRB and the HRPO is:

University of Maryland, Baltimore
Institutional Review Board
Human Research Protections Office
620 W. Lexington Street, Second Floor
Baltimore, MD 21201
410-706-5037

HP-00110284 UM IRB Approval Date 10/1/2024
Do Not Sign this Form after this Date 8/22/2025

<u>The myCircadianClock application</u>

As part of this study, you will use the myCircadianClock app to log some data and you may receive information and surveys through the app. The myCircadianClock app uses encrypted methods to transmit data between the app and the database, where the data are stored. The use of the app for research studies has been reviewed and approved by the Institutional Review Board of the Salk Institute. By consenting to this study, you are also consenting to use myCircadianClock app and also allowing the Salk research team to share data from the myCircadianClock app with our team.

- **myCircadianClock smartphone application ("app")**
  - The myCircadianClock app was created by and is managed by Dr. Satchidananda Panda's laboratory at the Salk Institute for Biological Studies. It is HIPAA-compliant and double-encrypted. Consenting to use and using the myCircadianClock app is necessary and critical for participation in the study.
- **Activities**
  - The myCircadianClock app may ask you to enter data about your lifestyle, including but not limited to: survey questions about your health behaviors, your body weight, what you eat or drink, exercise, and your sleep as best as you can.
  - The app sends occasional reminders to complete study activities.
  - Educational material may be sent through the app.
- **Sensor and health data**
  - This study can gather sensor data from your phone if you allow it to upon installation.
  - The myCircadianClock app may use the built-in accelerometer in certain phones to passively keep track of physical activity (passive because this happens automatically and you do not need to enter any information). The app interprets accelerometer data as steps taken, or as different intensity levels of activity. The app can also detect when you use the phone to get an independent estimate of your rest or sleep period. You can also sync the app to Apple Health Kit or Google Fit to capture activity, heart rate, and body temperature data.
  - For these activity measures to be accurate, you should carry the smartphone on your person as much as possible (e.g., in your pocket, or clipped to your waist). For instance, if the phone is left on a desk or in your car when you go for a walk, it will not be able to detect your walking.
  - The app will also use the built in GPS sensor to tag the location only when you log your data. This will help you to track what and where you eat, which may help you adjust your diet. It helps the research to account for any unusual change in your eating or sleeping pattern due to a change in time zone when you travel. Change in time zones or moving to a different latitude within the same time zone can change the local sunrise and sunset time or local day-length, which can affect your circadian clock for a few days.
  - <u>The use of the myCircadianClock app is not intended to evaluate your health and is not a diagnostic test. If you are concerned about any aspect of your health, you should consult with your physician.</u>

- **Data gathering**
    - Our daily pattern of activity, sleep, and eating changes with season, latitude, work schedule, and travel. Collected data may reveal how daily behaviors affect your quality of life and health, and the data will advance clinical research into circadian rhythms.
    - The myCircadianClock app helps you keep track of health behaviors such as diet, activity, sleep and taking your medications or supplements.
    - Data that you share through the app as part of the research study will create an unprecedented large-scale database of daily behaviors and health provided by people just like you.
    - Studying this real-world data will help researchers understand how daily behaviors influence health in real life, with a resolution never achieved before. (Traditionally, these studies are done by asking people to recall answers to very long questionnaires on paper).
    - At the same time, the myCircadianClock app analyzes your data to provide personalized insights into how your daily eating, sleeping and activity patterns relate to your overall health, and can help you maintain a healthy lifestyle. Because the feedback is determined from multiple days of your routine behavior, some of these insights will be accessible in your phone after a few days of data collection.
    - To improve data collection, the app may send you a reminder and push notifications. If you do not want to be bothered with these reminders, you can turn this option off.
    - By combining a personal app and a research study, myCircadianClock will help explore how the smartphone may be used with new kinds of clinical research in the future.
- **Privacy**
    - The following personal health information will be collected from you by the app: country, language, photos or names of food/beverages you take, activity/exercise, sleep, and health entries, timestamp of entries, and geographic location data from entries.
    - We take several steps to protect your privacy and the privacy of your app data.
    - Whenever app data is transferred to a research study computer, it will be encrypted so that others cannot interpret the data or associate it back to you.
    - Encrypted app data (stripped of personal identifiers, and associated only with a random code) will be sent to secure data servers used for the myCircadianClock research study.
    - Your encrypted data will be sent to a secure database where it will be stored with a unique identifier. The identifier does not contain any personal information. You will also receive your encrypted data back from the server for visualization on your phone.
    - App-generated data is associated only with a random participant code, and this code is used in all future analyses separating it from any personally identifiable information.
    - Study investigators chose Amazon Web Services for this important responsibility because they are a world leader in the secure storage and protection of sensitive data. They have a proven track record of safeguarding and managing potentially sensitive biomedical data in accordance with regulations that govern human research and medical information (e.g., regulations mandated by Institutional Review Boards [IRB] and the Health Information Portability and Accountability Act [HIPAA]).

- We will de-identify your data and use secure computers, but we cannot ensure complete privacy.
- One potential loss of privacy would be if someone sees your data from myCircadianClock on your smartphone. For security, myCircadianClock suggests that your smartphone be protected either by a passcode or fingerprint sensor. This ensures that only you can enter and use the app.
- These steps ensure that researchers analyzing the coded study data will not be able to connect it to any individual user.

- **Utilizing data**
  - The terms "study investigators" and "researchers" in this section refer to the research team who is conducting this study and the research team at the Salk Institute who oversee the use of the app.
  - Study investigators will analyze coded (no personal identifiers) app data from everyone who agrees to participate in myCircadianClock studies. Neither the data used for analysis nor the results will be able to be connected back to any individual user.
  - The results of this research may be published in a scientific or medical research journal so that others can learn from this study. Results will never be published in a way that would allow data to be associated with individual users.
  - Your coded data will be used for research and may be shared with other researchers.
  - After this study is completed, other researchers may request access to the coded study data (already stripped of personal identifiers), so that it can be analyzed in a new way to benefit medical research. Those requesting data must agree to use the data for research responsibly and in accordance with applicable regulations; these data requests will be reviewed by a group of study investigators. Amazon Web Services will have no oversight over future research conducted with coded study data.
  - Other researchers who are granted access to coded study data will not be able to connect the data back to you.
  - Study data will never be sold to any third party.
- **Issues to consider**
  - Logging food and sleep through the app for the study will take 5-10 minutes per day on average. Entering information and responding to surveys should take on average 2-3 minutes each day. Occasionally, tasks may take a few minutes longer (e.g., a longer questionnaire).
  - Participation in this study does not require you to change anything related to your smartphone account or data plan. However, your phone must have data or Wi-Fi capability and must connect to internet in order to transmit data to our servers. The app can use either an existing mobile data plan or Wi-Fi connections: you may configure the app to use only Wi-Fi connections if you wish to limit impact on your data usage. The study team or the Salk Institute bears no responsibility for bills related to your phone or data usage for participating in this study.

HP-00110284 UM IRB Approval Date 10/1/2024
Do Not Sign this Form after this Date 8/22/2025

- **Safety and legality**
  - As with any smartphone app, follow prevailing laws about when and where you use your smartphone. Similarly, follow local and federal regulations about the usage of a smartphone in specific areas.
  - Additionally, the app should not be used in any capacity to perform or document illegal activity.
  - The Salk Institute for Biological Studies, Dr. Satchidananda Panda and all members of his research team, including collaborators, are not liable for:
    - any illegal activity that is performed, captured, or stored by the myCircadianClock app.
    - any harm that may come from using the myCircadianClock app.
- **Study surveys**
  - For dietary information, myCircadianClock will prompt you to take pictures of every food, beverage, water, medication, and supplement(s) you take.
  - When picture taking is difficult or socially awkward as in a meeting, if you forgot to take picture or you are repeatedly eating the same item, you can also enter the information textually from a different screen.
  - In general, more data entered into the app results in more accurate and informative personalized insights.
- **Study tasks**
  - To gather additional information, including but not limited to activity (step counts/distance), heart rate, height and weight, you can sync other smartphone apps and sensory devices (such as Apple Health Kit and Google Fit) with myCircadianClock. You also have the choice to allow the myCircadianClock app to access your location in order to determine the local time. You can allow or deny these features when you first install the myCircadianClock app, and are able to change these settings at any time.
  - myCircadianClock has the option to add some information that requires a brief task away from the smartphone, such as: your weight, your height, your waist circumference, and your blood pressure. The app also has optional fields to enter relevant results from blood tests and urine samples including: blood glucose, lipid panels (total cholesterol, LDL, HDL, triglycerides), hemoglobin A1c, fibrinogen, C-reactive protein, homocysteine, and ketone bodies if you wish to monitor these parameters. Entering these data is optional.
  - <u>All data entered is used solely for tracking purposes, not diagnosis</u>. The Salk Institute for Biological Studies, Dr. Satchidananda Panda, and his lab are not responsible for providing medical advice and are not liable for your medical care. You should contact a medical professional for medical advice.
  - The myCircadianClock app may provide personalized feedback in the form of graphs and text to display your progress, and provide insights into your health behaviors.
  - The app may summarize data about how food, sleep, or activity patterns in a specific time of the day are associated with your health and wellbeing. These insights may help you

HP-00110284 UM IRB Approval Date 10/1/2024
Do Not Sign this Form after this Date 8/22/2025

understand your health behaviors better, and help you manage your health. Viewing the graphs and text is optional but may be useful or interesting to you.
    - For the passive sensing of your physical activity in some phone models to be accurate, it is important to carry your smartphone on your person (e.g., in your pocket, or clipped to your waist). Carrying your phone will also help you log food, beverage, water and activity data as soon as these events occur.
    - In your Profile within myCircadianClock, you can set reminders for yourself to complete app activities. In general, more data entered in the app results in more accurate and informative insights.
- **Meals, activity, and sleep:** The smartphone application myCircadianClock will serve as an electronic food, activity, and sleep diary.
    - On the server side, a sub-study dashboard will be created for this specific project. Clinical coordinators from Dr. Amber Kleckner's research team will have password-protected access to the study data. In the study summary dashboard, your study code and the date of activation of the app will be shown along with your daily log. If you fail to log any food data for more than 1 day, the dashboard flags you and sends an alert to the coordinator. The coordinators will login to the dashboard at least twice weekly to monitor food intake data, and follow up with you as necessary.
    - If you are randomized to the time-restricted eating arm, you will self-select an interval of 10 hours per day within which to consume your food. You can easily track your progress of the daily eating pattern with the time-stamping feature of the app that offers a visual summary.
    - If you have any difficulty logging data, or have questions about any of the features of the app, you will be able to contact the study coordinator through the feedback feature of the app. The questions will be delivered to a HIPAA-compliant email server specifically set up for this study.

HP-00110284 UM IRB Approval Date 10/1/2024
Do Not Sign this Form after this Date 8/22/2025

CONSENT TO PARTICIPATE – SIGNATURE PAGE

Signing this consent form indicates that you have read this consent form (or have had it read to you), that your questions have been answered to your satisfaction, and that you voluntarily agree to participate in this research study. You will receive a copy of this signed consent form.

RESEARCH PARTICIPANT

If you agree to participate in this study, please sign your name below.

Subject's Signature	Date	Printed Name

INVESTIGATOR OR DESIGNEE OBTAINING CONSENT

Signature of Investigator or Designee	Date	Printed Name

HP-00110284 UM IRB Approval Date 10/1/2024
Do Not Sign this Form after this Date 8/22/2025

HEALTH INSURANCE PORTABILITY AND ACCOUNTABILITY ACT (HIPAA)
AUTHORIZATION TO OBTAIN, USE, AND DISCLOSE
PROTECTED HEALTH INFORMATION FOR RESEARCH

Name of Study Subject: ____

Date of Birth: ________________ **Medical Record Number: ____________**

**NAME OF THIS RESEARCH STUDY:**	*Take the Reins: The effects of nutrient timing on cancer-related fatigue among blood cancer survivors*
**UMB IRB APPROVAL NUMBER:**	*HP-00110284*
**RESEARCHER'S NAME:**	*Amber Kleckner, PhD*
**RESEARCHER'S CONTACT INFORMATION:**	*Department of Pain and Translational Symptom Science*<br>*University of Maryland School of Nursing (SON)*<br>*655 W. Lombard Ave., Room 735*<br>*Baltimore MD 20201*<br>*410-706-5961*

This research study will use health information that identifies you. If you agree to participate, this researcher will use just the health information listed below.

THE SPECIFIC HEALTH INFORMATION TO BE USED OR SHARED:

- Demographics (e.g., height, weight, age, race, ethnicity, education, marital status)
- Details regarding cancer diagnosis and treatment (e.g., cancer site, cancer stage, chemotherapy type and dosing, surgical procedures, hormone therapy)
- Clinical characteristics (e.g., current menopausal status, Karnofsky Performance Status)
- Most recent blood work (e.g., hemoglobin, hematocrit, lymphocytes, etc.),
- Medical history (e.g., prior myocardial infarction, diabetes status).

Federal laws require this researcher to protect the privacy of this health information. She will share it only with the people and groups described here.

PEOPLE AND ORGANIZATIONS WHO WILL USE OR SHARE THIS INFORMATION:

- Dr. Amber Kleckner and the study team.
- The sponsor of the study or its agents, such as data repositories or contract research organizations
- Organization that will coordinate health care billing or compliance such as offices within the University of Maryland School of Nursing; the University of Maryland, Baltimore (UMB); University of Maryland Faculty Physicians, Inc. (FPI) and the faculty practices of the UMB; and University of Maryland Medical System (UMMS).

HP-00110284 UM IRB Approval Date 10/1/2024
Do Not Sign this Form after this Date 8/22/2025

THIS AUTHORIZATION WILL NOT EXPIRE, BUT YOU CAN REVOKE IT AT ANY TIME.

To revoke this Authorization, send a letter to this researcher stating your decision. The researcher will stop collecting health information about you. This researcher might not allow you to continue in this study. The researcher can use or share health information already gathered.

ADDITIONAL INFORMATION:

- You can refuse to sign this form. If you do not sign it, you cannot participate in this study. This will not affect the care you receive at:
  - University of Maryland Faculty Physicians, Inc. (FPI)
  - University of Maryland Medical System (UMMS)

  It will not cause any loss of benefits to which you are otherwise entitled.
- Sometimes, government agencies such as the Food and Drug Administration or the Department of Social Services request copies of health information. The law may require this researcher, the UMSOM, FPI, or UMMS to give it to them.
- This researcher will take reasonable steps to protect your health information. However, federal protection laws may not apply to people or groups outside the UMSOM, UMB, FPI, or UMMS.
- Except for certain special cases, you have the right to a copy of your health information created during this research study. You may have to wait until the study ends. Ask the researcher how to get a copy of this information from the study.

My signature indicates that I authorize the use and sharing of my protected health information for the purposes described above. I also permit my doctors and other healthcare providers to share my protected health information with this researcher for the purposes described above.

____

Subject's Signature   Date   Printed Name

Privacy Questions? Call the UMSOM Privacy Official (410-706-0337) with questions about your rights and protections under privacy rules.

Other Questions? Call the researcher named on this form with any other questions.

HP-00110284 UM IRB Approval Date 10/1/2024
Do Not Sign this Form after this Date 8/22/2025
