# Supplementary material for: Take the reins: a study protocol of a randomized controlled trial testing the effects of time-restricted eating vs. nutrition control on cancer-related fatigue among survivors of hematological malignancies
Source: BMC Nutr. 2025 Oct 31;11:200. doi: 10.1186/s40795-025-01185-0 (PMC12577101; doi:10.1186/s40795-025-01185-0)
Supplement: Supplementary file 2 — Supplementary Material 2. [file 40795_2025_1185_MOESM2_ESM.pdf]

FORMULARIO DE CONSENTIMIENTO PARA INVESTIGACIÓN Y AUTORIZACIÓN HIPAA

Título del protocolo: Tome las riendas: Efectos de la periodización de nutrientes sobre la fatiga relacionada con el cáncer en los supervivientes de un cáncer de la sangre

Número del estudio: HP-00110284

Investigador principal: Amber Kleckner, PhD, 410-706-5961

Patrocinador: Instituto Nacional del Cáncer (NIH) e Instituto Nacional de Salud (NCI)

Este documento de consentimiento describe un estudio de investigación, lo que puede esperar si decide participar y la información importante que le ayudará a tomar una decisión. Lea atentamente este formulario y haga las preguntas que desee antes de acceder a participar.

RESUMEN BREVE:

La mayoría de los pacientes experimentan cansancio durante el tratamiento del cáncer, lo que se denomina "fatiga relacionada con el cáncer". Para muchas personas, la fatiga no desaparece después del tratamiento. Los médicos y científicos no comprenden totalmente por qué se produce la fatiga, y no disponemos de buenas formas de tratarla. Hay nuevos datos que indican que la fatiga podría deberse a que el reloj corporal de una persona (es decir, el ritmo circadiano) está desajustado. Sin embargo, esto no se ha demostrado. En este estudio, recopilaremos datos sobre las actividades cotidianas relacionadas con los ritmos biológicos (patrones de sueño, actividad física y dieta) para obtener más información sobre las relaciones entre el ritmo circadiano y la fatiga.

El estudio durará aproximadamente 14 semanas, con un seguimiento de 24 semanas. Al comienzo del estudio, se le pedirá que responda a cuestionarios en línea relacionados con sus síntomas y sentimientos. Durante una semana, le pediremos que registre su dieta y sueño mediante una aplicación para teléfonos inteligentes llamada myCircadianClock. Durante la misma semana, le pediremos que lleve un monitor de actividad en la muñeca (como un reloj) y un monitor continuo de glucosa en la parte posterior del brazo. Después de la primera semana, todos se reunirán con un nutricionista para hablar de la nutrición en la supervivencia del cáncer. Se le asignará aleatoriamente a uno de dos grupos: se pedirá a un grupo que ingiera toda la comida dentro de un periodo de 10 horas durante el día por 12 semanas (usted elige la hora de inicio, por ejemplo, entre las 9:00 a. m. y las 7:00 p. m.); al otro grupo no se le pedirá que cambie *de hora* de comer. A la mitad (semana 6) y al final (semana 12) del estudio, le pediremos de nuevo que realice las mismas actividades del estudio que al principio (cuestionarios, registro de la dieta y el sueño, uso del monitor de actividad y monitor continuo de glucosa). Después de 24 semanas, le pediremos que responda a una serie adicional de cuestionarios. Los materiales del estudio podrán entregarse en persona o por correo postal, a su elección (es decir, no tendrá que acudir al centro). Se le pagará un total de $100 por el tiempo dedicado para llevar a cabo las actividades del estudio.

HP-00110284 UM IRB Approval Date 1/24/2025
Do Not Sign this Form after this Date 8/22/2025

Principales riesgos: malestar por el monitor de actividad o el monitor de glucosa; angustia emocional; pérdida de confidencialidad; pérdida de peso, molestias digestivas o hambre por alimentación con límite de tiempo

La participación en este estudio es voluntaria. Su decisión de participar no afectará en modo alguno su asistencia médica ni el tratamiento del cáncer.

PROPÓSITO DEL ESTUDIO

En este estudio, queremos obtener más información sobre las relaciones entre los comportamientos relacionados con el modo de vida (patrones de sueño, actividad física y dieta) y la fatiga. La "alimentación con límite de tiempo" es cuando se consume toda la comida en un período bien definido durante el día. Comprobaremos si la alimentación con límite de tiempo (un período de 10 horas), en comparación con un período de alimentación más largo, modifica el grado de fatiga de la persona.

Se reclutará a unos 100 participantes para participar en este estudio realizado en el University of Maryland Medical System.

PROCEDIMIENTOS

Estas son las actividades y el cronograma del estudio. Cada una de las actividades se describe con más detalle a continuación.

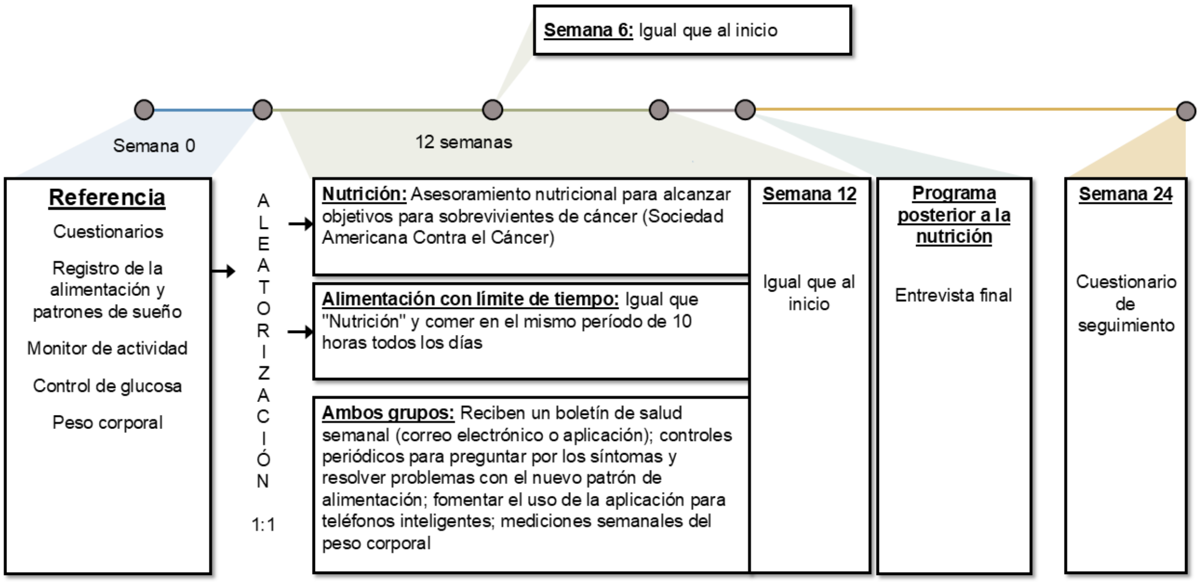

<u>Cuestionarios:</u> Le pediremos que responda a varios cuestionarios en línea sobre sus síntomas, sentimientos y hábitos. Tardará de 30 a 60 minutos en completarlos. También tiene la opción de hacerlo en papel. Le pediremos que responda los cuestionarios cuatro veces durante todo el estudio, en las semanas 0, 6, 12 y 24.

HP-00110284 UM IRB Approval Date 1/24/2025
Do Not Sign this Form after this Date 8/22/2025

Registro de alimentación y patrones de sueño: Le pediremos que registre todo lo que come cada día usando la aplicación myCircadianClock en las semanas 0, 6 y 12. Además, le pediremos que registre cuándo acuesta y cuándo se levanta usando la aplicación. El registro de los patrones de alimentación y sueño es opcional en las semanas 1 a 5 y 7 a 11. Esta actividad durará unos 5 minutos al día. También tiene la opción de anotar el consumo de alimentos en papel.

Actigrafía: Le pediremos que lleve un monitor de actividad en la muñeca todos los días durante las semanas 0, 6 y 12. El monitor de actividad controla la actividad física.

Control de glucosa: Le pediremos que lleve un monitor continuo de glucosa todos los días en las semanas 0, 6 y 12. Este mide la *glucosa intersticial*, que es la concentración de azúcar entre las células y se correlaciona estrechamente con el azúcar en sangre. El monitor continuo de glucosa tiene un tamaño de dos monedas de 25 centavos de dólar apiladas una encima de otra. Se coloca en la parte posterior del brazo. Tiene un alambre fino de 5 mm de longitud que se inserta bajo la piel y es resistente al agua para que no le moleste. Se coloca una vez al principio de la semana y puede mantenerlo durante 14 días como máximo, pero solo le pedimos que lo lleve puesto durante 7 días. Podemos colocarlo en su lugar en el Centro Médico, o podemos enviárselo por correo para que usted mismo se lo coloque. Le enseñaremos cómo colocarse el monitor y estaremos a su disposición si tiene alguna duda cuando lo haga. Al principio se siente como un pellizco pequeño.

Peso corporal: Le proporcionaremos una báscula digital de baño que puede usar en su casa. Pésese una vez por semana e ingrese el peso en la aplicación myCircadianClock o en un registro en papel.

Aleatorización y los dos grupos del estudio: Después de la primera semana, todos se reunirán con un nutricionista para hablar de la nutrición en la supervivencia del cáncer. Se le asignará aleatoriamente a uno de dos grupos: el grupo de alimentación con límite de tiempo o el grupo de alimentación *sin* límite de tiempo. Nadie del equipo del estudio sabe en qué grupo estará hasta después de la semana 0. El tratamiento que reciba será elegido al azar, como cuando se echa una moneda al aire. Hay un 50 % de probabilidades de que esté en el grupo de alimentación con límite de tiempo y un 50 % de probabilidades de que esté en el grupo de alimentación sin límite de tiempo.

- Alimentación con límite de tiempo: Si se le asigna al grupo de alimentación con límite de tiempo, elegirá un periodo de 10 horas para comer según sus horarios y preferencias. Por ejemplo, este periodo podría comenzar a las 7:00 a. m., 9:15 a. m. u otra hora. Podrá beber agua en cualquier momento. Se permite el té sin endulzar y el café negro por la mañana antes de la hora de comer. ***Todos los demás alimentos y bebidas, incluidos los edulcorantes artificiales (p. ej., chicle o refrescos dietéticos), podrán consumirse solo dentro del periodo de 10 horas.*** Dentro del periodo de 10 horas, podrá comer lo que desee en cualquier momento, aunque se le anima a cumplir los objetivos que hable con el nutricionista.
- Alimentación sin límite de tiempo: Si es asignado al grupo de alimentación sin límite de tiempo, debe cumplir con las recomendaciones acordadas con el nutricionista sin restricción en *cuanto a la hora* de comer.

Contacto con el equipo del estudio: Le llamaremos aproximadamente cada dos semanas para comprobar cómo se encuentra. También puede llamar y enviar un correo electrónico al equipo del estudio entre estas comprobaciones.

Aplicación myCircadianClock: Además de usar la aplicación para registrar los patrones de alimentación, los patrones de sueño y el peso corporal, todos los participantes en el estudio recibirán consejos semanales sobre estilos de vida saludables a través de la aplicación.

Entrevista final: Cuando finalice el estudio, o si decide retirarse prematuramente, le "entrevistaremos" acerca de su experiencia en el estudio. Le preguntaremos qué le ha gustado de este, qué no le ha gustado y le pediremos su opinión sobre la aplicación myCircadianClock. Si estaba en el grupo de alimentación con restricción de tiempo, le preguntaremos sobre sus experiencias con el nuevo patrón de dieta. Esta entrevista durará entre 20 y 30 minutos y grabaremos el audio de la conversación (no el video) si le parece bien.

Todas estas actividades de la investigación pueden hacerse a distancia. Sin embargo, le invitamos a venir y le ayudaremos a colocarse el monitor de glucosa, usar la aplicación myCircadianClock, completar cuestionarios o llevar a cabo las otras actividades del estudio. Estamos en la Escuela de Enfermería, frente al Greenebaum Comprehensive Cancer Center.

¿CUÁLES SON MIS RESPONSABILIDADES SI PARTICIPO EN ESTE ESTUDIO DE INVESTIGACIÓN?

Si participa en este estudio, será responsable de participar en las actividades del estudio, tal como se han descrito anteriormente.

POSIBLES RIESGOS Y MOLESTIAS:

Como en todos los estudios de investigación, existen riesgos asociados a las actividades del estudio. Hemos adoptado medidas para reducir al mínimo todos los riesgos previstos. Tenga en cuenta estos riesgos cuando decida si desea participar.

1. *Molestias debidas al monitor continuo de glucosa*
   El monitor continuo de glucosa es un dispositivo mínimamente invasivo que detecta el azúcar justo debajo de la piel. Se aplica en la parte posterior del brazo y se siente como un pellizco cuando se inserta el sensor pequeño de 5 mm de longitud. También puede causar dolor si el dispositivo recibe un golpe mientras lo lleva puesto. Elegimos utilizar este dispositivo en lugar de obtener mediciones de la glucemia porque evita los pinchazos dolorosos en el dedo.

2. *Angustia emocional*
   Podría sentirse molesto o abrumado por la expectativa de consumir alimentos solo en el periodo de alimentación. Sin embargo, queremos destacar que no está "en problemas" si no sigue exactamente los procedimientos. Queremos utilizar lo que aprendamos de este estudio para mejorar los procedimientos del siguiente.

   Nuestros cuestionarios contienen información que puede generar inquietud o ser privada (p. ej., "Estoy satisfecho con la comunicación sobre mi enfermedad con mi familia"). No tiene que responder a ninguna pregunta que le resulte incómoda y puede tomarse un descanso o dejar de responder a los cuestionarios en cualquier momento.

HP-00110284 UM IRB Approval Date 1/24/2025
Do Not Sign this Form after this Date 8/22/2025

3. *Infracción de la confidencialidad*
Siempre existe el riesgo de que se infrinja la confidencialidad cuando personas ajenas al equipo de investigación puedan llegar a conocer información médica sensible. Para evitar la fuga de información confidencial, solo la Dra. Amber Kleckner (la directora del estudio), Carin Clingan (la coordinadora del estudio) y las personas designadas tendrán acceso al registro de selección y al archivo que vincula su nombre con su número de sujeto (ambos estarán cifrados); estos archivos se almacenarán en computadoras protegidas con contraseña en sus oficinas privadas. Todos los archivos de datos harán referencia a usted mediante una identificación de participante no identificable y se almacenarán en la computadora de la Dra. Kleckner y en servidores seguros de la UMB. Todos los documentos de consentimiento se guardarán en un armario cerrado con llave, también en el consultorio de su personal. Todas las entrevistas grabadas en audio se transferirán al ordenador y al servidor seguros de la Dra. Kleckner en la UMB en el plazo de 2 días laborables desde la entrevista y se eliminarán inmediatamente de la grabadora. Los nombres de los archivos de las entrevistas no incluirán su nombre ni ninguna información identificativa. Si la Dra. Kleckner comparte datos con cualquier otro investigador para los análisis, se eliminará la identificación de todos los datos (es decir, no tendrá su nombre, fecha de nacimiento, información de contacto, etc.). La presentación de los resultados del estudio en forma de presentaciones y manuscritos, ya sea en entornos privados o públicos, no tendrá ninguna información identificable, ni se reproducirán en público clips de audio. La Dra. Kleckner y el resto de colaboradores participan en la capacitación ética de acuerdo a las políticas institucionales.

4. *Pérdida de peso por la alimentación con límite de tiempo*
Este no es un estudio sobre la pérdida de peso. Algunos estudios han documentado la pérdida de peso con la adopción de un nuevo patrón de alimentación, a pesar de no "reducir las calorías" a propósito. Le animamos a comer y beber para saciar el hambre y la sed. Sin embargo, cambiará sus patrones de alimentación y existe la posibilidad de que pierda peso durante el estudio. La pérdida de peso lenta no se considerará peligrosa (menos del 3 % del peso corporal por semana), especialmente si empieza el estudio con sobrepeso. Sin embargo, si experimenta una pérdida de peso rápida y no intencionada, según lo determinado por un profesional médico, él le aconsejará sobre lo que debe hacer, lo que podría suponer la retirada del estudio.

5. *Malestar gastrointestinal*
Con un cambio en la dieta, y especialmente uno importante, podría experimentar estreñimiento, diarrea, náuseas, ardor de estómago, etc. Estos efectos suelen desaparecer después de varios días, pero siempre tendrá acceso al equipo del estudio para preguntar cómo aliviar estos efectos.

6. *Hambre o hipoglucemia*
Los que pertenezcan al grupo de alimentación con límite de tiempo tomarán su comida en un período más corto de lo normal. Si le asignan a este grupo, puede tener hambre cuando esté fuera del periodo de alimentación. El hambre puede acompañarse de fatiga, falta de concentración, irritabilidad, dolor de cabeza y otros síntomas. Es probable que estos síntomas desaparezcan al cabo de unos días, cuando el organismo se adapta al nuevo patrón. Sin embargo, los síntomas moderados o graves pueden ser un signo de hipoglucemia (nivel bajo de azúcar en sangre) y le recomendamos que coma fuera de periodo si se siente aletargado, mareado o enfermo.

HP-00110284 UM IRB Approval Date 1/24/2025
Do Not Sign this Form after this Date 8/22/2025

7. *Pérdida de la privacidad*
   Nuestro equipo del estudio accederá a su historia clínica y hablará con usted sobre cuestiones que podría considerarse privadas. No es necesario que responda a ninguna pregunta que le incomode.

POSIBLES BENEFICIOS

No es seguro que vaya a obtener algún beneficio por participar en este estudio de investigación. Es posible que tome más conciencia de su dieta y patrones de sueño, lo que puede contribuir a la salud en general. Sin embargo, no hay garantías de que recibirá un beneficio directo de este estudio.

ALTERNATIVAS A LA PARTICIPACIÓN

Este no es un estudio de tratamiento y la alternativa es no participar. En lugar de participar, puede optar por registrar su dieta y patrones de sueño por su cuenta o acudir a un médico (p. ej., dietista o médico de atención primaria) para que examine cómo su dieta, actividad física y patrones de sueño pueden estar contribuyendo a su fatiga. Si decide no participar en el estudio, su decisión de no participar no afectará a su asistencia médica.

COSTOS PARA LOS SUJETOS

Para inscribirse en el estudio no hay que pagar una tarifa. También puede incurrir en costos complementarios, como desplazamientos, alojamiento, estacionamiento, alimentación, etc.

PAGO A LOS SUJETOS

Se le pagará un total de $100 por el tiempo dedicado a llevar a cabo las actividades del estudio. Le pagaremos un total de $100 para llevar a cabo todas las actividades del estudio: $25 cada una para las semanas 0, 6 y 12, que se le pagarán después de la semana 12 o se prorratearán si decide retirarse del estudio antes de tiempo. Después de la semana 24, recibirá otros $25 por responder los cuestionarios. Estos pagos se hacen mediante tarjetas de regalo electrónicas o mediante cheque.

Es posible que tenga que declarar los pagos que recibe por participar en el estudio como ingresos gravados, lo que podría afectar su elegibilidad para recibir ciertos beneficios gubernamentales (por ejemplo, del Programa de Asistencia Nutricional Suplementaria (SNAP) de Maryland y el Programa de Asistencia de Efectivo Temporal (TCA) de Maryland). Si tiene una deuda con el estado de Maryland o con el gobierno federal (por ejemplo, manutención infantil o impuestos), es posible que se reduzca el monto que recibe.

LESIONES RELACIONADAS CON EL ESTUDIO

Si tiene una lesión, busque atención médica rápidamente de cualquier proveedor de atención médica. <u>Si tiene una emergencia, llame al 911 o acuda a la sala de urgencias más cercana.</u> Debe informar al proveedor de atención médica que ha participado en un estudio de investigación.

Si considera que la lesión está relacionada con el estudio, informe al médico del estudio. La Universidad de Maryland, Baltimore (UMB), si se lo solicita, le ayudará a obtener atención médica o remisiones.

HP-00110284 UM IRB Approval Date 1/24/2025
Do Not Sign this Form after this Date 8/22/2025

Si sufre algún daño como consecuencia de su participación en este estudio, usted o su seguro serán los responsables de pagar los gastos médicos. Ni el hospital ni la UMB han aceptado pagar el costo de la asistencia médica u otros costos derivados de una lesión. Sin embargo, no renuncia a ninguno de sus derechos legales por participar en este estudio y puede optar por emprender acciones legales si sufre algún daño por participar en este.

CONFIDENCIALIDAD Y ACCESO A REGISTROS

Con su número de historia clínica, accederemos a su historia clínica electrónica para obtener información sobre su diagnóstico y antecedentes de tratamiento del cáncer, historia clínica e historia social. Recopilaremos su nombre, dirección, número de teléfono y dirección de correo electrónico para comunicarnos con usted con el fin de programar y recordar las próximas actividades del estudio. Solo la Dra. Amber Kleckner, la investigadora principal y el personal de investigación formado y designado tendrán acceso a la información confidencial. Toda la información confidencial que incluya información de identificación personal se codificará con un número de identificación del estudio. La investigadora principal y la coordinadora del estudio serán las únicas personas con acceso a la clave de los números de identificación asignados. Toda la información confidencial se guardará bajo llave en un gabinete en un lugar seguro en la Facultad de Enfermería de la Universidad de Maryland. Su información de identificación personal no se utilizará para los análisis de este estudio, pero se conservará en el archivo si los organismos federales o el Comité de Revisión Institucional (CRI) tienen la obligación de revisar cualquier información.

Todos los registros del estudio se considerarán confidenciales y no se utilizarán en informes ni publicaciones los nombres ni la información de identificación personal de los participantes. Se hará todo lo posible por limitar la divulgación de información personal, incluso los registros del estudio de investigación y las historias clínicas, a las personas que tengan la necesidad de revisar esta información. No podemos prometer total privacidad. Las organizaciones que podrían inspeccionar y copiar su información incluyen el CRI, otros representantes de la Universidad de Maryland, Baltimore (UMB) o el centro del estudio. Los monitores, auditores, el CRI y la Administración de Alimentos y Medicamentos tendrán acceso directo a su historia clínica para verificar los procedimientos y las fechas del estudio de investigación. Al firmar este documento autoriza dicho acceso. Las personas designadas por la Universidad de Maryland podrán examinar determinados registros de investigación de este estudio; sin embargo, cualquier persona que inspeccione esta información deberá mantener la confidencialidad de esta información personal. Su información personal no se divulgará, salvo que lo exija la ley. Al firmar este documento, autoriza a los monitores, auditores y al CRI a tener acceso a esta información.

Los datos provenientes del estudio pueden ser publicados. Sin embargo, no será identificado por su nombre. Las personas designadas de las instituciones donde se lleva a cabo el estudio y el personal del Patrocinador tendrán permiso para inspeccionar partes de su historia clínica y registros de la investigación en relación con el estudio. Todas las personas que usan la información del estudio se esforzarán por mantener la confidencialidad de su información personal. Su información personal no se divulgará, salvo que lo exija la ley.

En http://www.clinicaltrials.gov, estará disponible una descripción de este ensayo clínico, tal como lo exige la ley de los EE. UU. Este sitio web no incluye información que pueda identificarlo. A lo sumo, el sitio web incluirá un resumen de los resultados. Puede hacer búsquedas en este sitio web en cualquier momento.

HP-00110284 UM IRB Approval Date 1/24/2025
Do Not Sign this Form after this Date 8/22/2025

Estamos utilizando la aplicación myCircadianClock para recopilar datos sobre su consumo de alimentos, patrones de sueño y peso corporal. La aplicación myCircadianClock fue desarrollada por investigadores del Instituto Salk en La Jolla, California, y ha sido utilizada en otros estudios de investigación. Al final de este documento encontrará información detallada sobre la aplicación. **No compartiremos ninguna información personal (p. ej., su nombre) ni información médica (p. ej., detalles de su diagnóstico de cáncer) con el equipo de myCircadianClock, aunque tendrán acceso a cualquier información que usted ingrese en la aplicación.** Al otorgar su consentimiento para este estudio, acepta que sus datos sin identificación obtenidos de la aplicación se almacenen en el Salk Institute y se combinen con otros datos de myCircadianClock en futuros análisis. Al final de este estudio, podrá solicitar que se eliminen sus datos de su base de datos.

DERECHO A RETIRARSE

Su participación en este estudio es voluntaria. No está obligado a participar en este estudio de investigación. Es libre de retirar su consentimiento en cualquier momento. Su decisión de no participar o interrumpir la participación en el estudio no implica sanciones ni pérdida de los beneficios que le corresponden de cualquier otra manera. Si decide dejar de participar, o si tiene preguntas, inquietudes o quejas, o si necesita reportar una lesión relacionada con el estudio, comuníquese con la investigadora, la Dra. Amber Kleckner, al teléfono 410-706-5961. Para suspender su participación en el estudio, debe solicitar el retiro por escrito enviándolo a la Dra. Amber Kleckner a amber.kleckner@umaryland.edu.

Si se retira de este estudio, es posible que los datos ya recopilados no sean eliminados de la base de datos del estudio. Se le preguntará si el investigador puede recopilar los datos provenientes de su atención médica habitual. Si acepta, estos datos serán tratados de la misma manera que los datos de la investigación.

Se le informará cualquier hallazgo significativo nuevo que se produzca durante el estudio y que pueda afectar su voluntad de participar en el estudio.

¿PUEDEN RETIRARME DEL ESTUDIO?

La persona a cargo del estudio o el patrocinador pueden excluirlo de este sin su aprobación. Entre los posibles motivos del retiro se incluyen la imposibilidad de comunicarse con usted, si su salud empeora o si el médico considera que su permanencia en el estudio es perjudicial para su salud o si su participación resulta perjudicial para un miembro del equipo del estudio. El patrocinador también puede finalizar el estudio de manera anticipada. El equipo del estudio le informará al respecto y podrá hacer preguntas si esto ocurre.

DECLARACIÓN DE LA UNIVERSIDAD

La Universidad de Maryland, Baltimore (UMB) se compromete a proporcionar a los sujetos de sus estudios de investigación todos los derechos que les corresponden conforme a la ley estatal y federal. Usted no renuncia a ninguno de sus derechos legales por firmar este formulario de consentimiento ni por participar en este estudio. Este estudio fue revisado y aprobado por el Comité de Revisión Institucional (CRI). El CRI es un grupo de científicos, médicos, expertos y representantes de la comunidad. La

HP-00110284 UM IRB Approval Date 1/24/2025
Do Not Sign this Form after this Date 8/22/2025

membresía del CRI incluye a personas que no están afiliadas con la UMB y personas que no realizan estudios de investigación.

Si tiene preguntas, inquietudes, quejas o considera que ha resultado perjudicado por su participación en este estudio de investigación como resultado de la negligencia del investigador, puede comunicarse con los miembros del CRI o la Oficina de Protección de los Seres Humanos en la Investigación (HRPO) para hacer preguntas, comentar problemas o inquietudes, obtener información u ofrecer información sobre sus derechos como sujeto de un estudio de investigación. La información de contacto del CRI y la Oficina de Protección de la Investigación en Seres Humanos (HRPO) es:

Universidad de Maryland, Baltimore
Comité de Revisión Institucional
Oficina para la Protección de Seres Humanos en Estudios de Investigación
620 W. Lexington Street, Second Floor
Baltimore, MD 21201
410-706-5037

HP-00110284 UM IRB Approval Date 1/24/2025
Do Not Sign this Form after this Date 8/22/2025

La aplicación myCircadianClock

Como parte de este estudio, usará la aplicación myCircadianClock para registrar algunos datos y es posible que reciba información y encuestas a través de esta. La aplicación myCircadianClock usa métodos cifrados para transmitir datos entre la aplicación y la base de datos, donde se almacenan los datos. El uso de la aplicación para estudios de investigación ha sido revisado y aprobado por el Comité de Revisión Institucional del Salk Institute. Al dar su consentimiento para este estudio, también da su consentimiento para usar la aplicación myCircadianClock y también permite que el equipo de investigación de Salk comparta los datos de la aplicación myCircadianClock con nuestro equipo.

- **Aplicación para teléfonos inteligentes myCircadianClock (la "aplicación")**
  - La aplicación myCircadianClock fue creada y es administrada por el laboratorio de la Dra. Satchidananda Panda en el Salk Institute for Biological Studies. Cumple con la HIPAA y está doblemente cifrada. Es necesario y fundamental que dé su consentimiento para usar y usar la aplicación myCircadianClock a fin de participar en el estudio.
- **Actividades**
  - La aplicación myCircadianClock puede pedirle que ingrese datos sobre su estilo de vida, incluidos, entre otros: preguntas de la encuesta sobre sus comportamientos de salud, su peso corporal, lo que come o bebe, ejercicio y su sueño lo mejor que pueda.
  - La aplicación envía recordatorios ocasionales para que lleve a cabo las actividades del estudio.
  - Puede enviarse material educativo mediante la aplicación.
- **Sensor y datos de salud**
  - Este estudio puede recopilar datos del sensor del teléfono si lo permite al instalarlo.
  - La aplicación myCircadianClock puede usar el acelerómetro incorporado en ciertos teléfonos para llevar un registro pasivo de la actividad física ("pasivo" quiere decir que sucede automáticamente y no es necesario ingresar ninguna información). La aplicación interpreta los datos del acelerómetro como pasos dados o como diferentes niveles de intensidad de la actividad. La aplicación también puede detectar cuándo utiliza el teléfono para hacer un cálculo independiente de sus períodos de descanso o sueño. También puede sincronizar la aplicación con Apple Health Kit o Google Fit para capturar datos de actividad, frecuencia cardíaca y temperatura corporal.
  - Para que estas medidas de actividad sean exactas, deberá llevar el teléfono inteligente lo más cerca posible (por ejemplo, en el bolsillo o sujeto a la cintura). Por ejemplo, si deja el teléfono en el escritorio o en su automóvil cuando sale a caminar, no podrá detectar sus pasos.
  - La aplicación también utilizará el sensor GPS incorporado para etiquetar la ubicación solo cuando registra sus datos. Esto le ayudará a llevar un registro de lo que come y dónde lo come, lo que puede ayudarle a ajustar su dieta. Ayuda a la investigación a explicar cualquier cambio inusual en su patrón de alimentación o sueño debido a un cambio en la zona horaria cuando viaja. El cambio de zona horaria o el cambio a una latitud diferente dentro de la misma zona horaria puede cambiar la hora local del amanecer y el atardecer o la duración del día local, lo que puede afectar el reloj circadiano durante unos días.

HP-00110284 UM IRB Approval Date 1/24/2025
Do Not Sign this Form after this Date 8/22/2025

- El uso de la aplicación myCircadianClock no tiene como fin evaluar su salud y no es una prueba diagnóstica. Si le preocupa algún aspecto de su salud, consulte a su médico.

- **Recopilación de datos**
    - Nuestro patrón diario de actividad, sueño y alimentación cambia con la estación, la latitud, el horario de trabajo y los viajes. Los datos recopilados pueden revelar cómo los comportamientos diarios afectan la calidad de su vida y salud, y los datos apoyarán la investigación clínica de los ritmos circadianos.
    - La aplicación myCircadianClock le ayuda a llevar un registro de los comportamientos relacionados con la salud, como dieta, actividad, sueño y toma de medicamentos o suplementos.
    - Los datos que comparta a través de la aplicación como parte del estudio de investigación crearán una base de datos a gran escala sin precedentes de comportamientos y salud diarios proporcionados por personas como usted.
    - El estudio de estos datos de la vida real ayudará a los investigadores a entender cómo influyen las conductas diarias en la salud en la vida real, con una resolución nunca alcanzada antes. (Tradicionalmente, estos estudios se realizan pidiendo a las personas que recuerden las respuestas respondiendo cuestionarios impresos muy largos).
    - Al mismo tiempo, la aplicación myCircadianClock analiza sus datos para proporcionar información personalizada sobre cómo sus patrones diarios de alimentación, sueño y actividad se relacionan con su salud general, y puede ayudarle a mantener un estilo de vida saludable. Dado que la retroalimentación se determina a partir de varios días de su comportamiento habitual, algunas de estas percepciones serán accesibles en su teléfono después de unos días de recopilación de datos.
    - Para mejorar la recolección de datos, la aplicación puede enviarle un recordatorio y notificaciones automáticas. Si no desea que le molesten estos recordatorios, puede desactivar esta opción.
    - Al combinar una aplicación personal y un estudio de investigación, myCircadianClock ayudará a explorar cómo puede utilizarse los teléfonos inteligentes con nuevos tipos de investigación clínica en el futuro.
- **Privacidad**
    - La aplicación recopilará la siguiente información médica personal: país, idioma, fotografías o nombres de los alimentos o bebidas que tome, actividad o ejercicio, anotaciones sobre el sueño y la salud, hora y localización geográfica de los registros.
    - Tomamos varias medidas para proteger su privacidad y la privacidad de los datos de su aplicación.
    - Siempre que los datos de la aplicación se transfieran a una computadora del estudio de investigación, se codificarán de forma que otras personas no puedan interpretar los datos ni relacionarlos con usted.
    - Los datos de aplicaciones cifrados (desprovistos de identificadores personales y asociados únicamente con un código aleatorio) se enviarán a servidores de datos seguros utilizados para el estudio de investigación myCircadianClock.

- Sus datos cifrados se enviarán a una base de datos segura donde se conservarán con un identificador único. Este identificador no contiene ninguna información personal. También recibirá sus datos cifrados del servidor para su visualización en el teléfono.
- Los datos generados por la aplicación se asocian únicamente a un código de participante aleatorio y este código se utiliza en todos los análisis futuros que lo separan de cualquier información de identificación personal.
- Los investigadores del estudio eligieron Amazon Web Services por esta importante responsabilidad porque son líderes mundiales en el almacenamiento seguro y la protección de datos confidenciales. Cuentan con una trayectoria demostrada de protección y gestión de datos biomédicos potencialmente sensibles de conformidad con las normas que rigen la investigación humana y la información médica (p. ej., normas exigidas por los comités de revisión institucional [CRI] y la Ley de Transferencia y Responsabilidad de Seguro Médico [HIPAA]).
- Eliminaremos la identificación de sus datos y usaremos computadoras seguras, pero no podemos garantizar una privacidad completa.
- Una posible pérdida de privacidad sería si alguien viera sus datos de myCircadianClock en su teléfono inteligente. Por seguridad, myCircadianClock sugiere que su teléfono inteligente esté protegido con un código de acceso o un lector de huellas digitales. Esto garantiza que solo usted pueda entrar y utilizar la aplicación.
- Estos pasos garantizan que los investigadores que analicen los datos codificados del estudio no puedan conectarlos a ningún usuario individual.

- **Uso de los datos**
  - Los términos "investigadores del estudio" e "investigadores" en esta sección se refieren al equipo de investigación que realiza este estudio y al equipo de investigación del Salk Institute que supervisa el uso de la aplicación.
  - Los investigadores del estudio analizarán los datos codificados de la aplicación (sin identificadores personales) de todas las personas que acepten participar en los estudios myCircadianClock. Ni los datos utilizados para el análisis ni los resultados podrán conectarse de nuevo a ningún usuario individual.
  - Los resultados de esta investigación podrán publicarse en revistas científicas o médicas para que otras personas puedan aprender de este estudio. Los resultados nunca se publicarán de un modo que permita asociar los datos con usuarios individuales.
  - Sus datos codificados se utilizarán para fines de investigación y podrán compartirse con otros investigadores.
  - Una vez finalizado este estudio, otros investigadores podrán solicitar el acceso a los datos codificados del estudio (ya desprovistos de identificadores personales) para poder analizarlos de una nueva forma que beneficie a la investigación médica. Quienes soliciten los datos deberán comprometerse a utilizarlos para fines de investigación de forma responsable y de conformidad con la normativa vigente; estas solicitudes de datos serán revisadas por un grupo de investigadores del estudio. Amazon Web Services no supervisará las investigaciones futuras realizadas con datos codificados del estudio.

- Otros investigadores que tengan acceso a los datos codificados del estudio no podrán relacionarlos con usted.
- Los datos del estudio nunca se venderán a terceros.

- **Aspectos a tener en cuenta**
  - Registrar la comida y el sueño a través de la aplicación del estudio le llevará entre 5 y 10 minutos al día en promedio. La entrada de información y respuestas de las encuestas deben llevarle de 2 a 3 minutos al día en promedio. En ocasiones, las tareas pueden llevarle unos minutos más (p. ej., un cuestionario más largo).
  - La participación en este estudio no requiere que cambie nada relacionado con su cuenta de teléfono inteligente o plan de datos. Sin embargo, su teléfono debe tener datos o capacidad Wi-Fi y debe conectarse a Internet para transmitir datos a nuestros servidores. La aplicación puede utilizar un plan de datos móviles existente o conexiones Wi-Fi: puede configurar la aplicación para que utilice únicamente conexiones Wi-Fi si desea limitar el impacto en el uso de datos. El equipo del estudio o el Instituto Salk no son responsables de las facturas relacionadas con su teléfono o el uso de los datos para participar en este estudio.
- **Seguridad y legalidad**
  - Al igual que con cualquier aplicación para teléfonos inteligentes, respete las leyes vigentes sobre cuándo y dónde utiliza su teléfono inteligente. Del mismo modo, siga las normas locales y federales sobre el uso de teléfonos inteligentes en áreas específicas.
  - Además, la aplicación no debe utilizarse en ninguna capacidad para realizar o documentar actividades ilegales.
  - El Instituto Salk de Estudios Biológicos, el Dr. Satchidananda Panda y todos los miembros de su equipo de investigación, incluyendo colaboradores, no son responsables de:
    - cualquier actividad ilegal que realice, capture o almacene la aplicación myCircadianClock.
    - cualquier perjuicio que pueda derivarse del uso de la aplicación myCircadianClock.
- **Encuestas de estudio**
  - Para obtener información de la dieta, myCircadianClock le pedirá que tome fotografías de todos los alimentos, bebidas, agua, medicamentos y suplementos que tome.
  - Cuando tomar las fotos sea difícil o socialmente incómodo, como durante una reunión, o si olvidó tomar una foto o está comiendo repetidamente el mismo alimento, también puede ingresar la información textualmente desde otra pantalla.
  - En general, entre más datos se introduzcan en la aplicación se producirá más información personalizada más precisa e informativa.
- **Tareas del estudio**
  - Para recopilar información adicional, que incluye, entre otras cosas, actividad (cantidad de pasos o distancia), frecuencia cardíaca, estatura y peso, puede sincronizar otras aplicaciones para teléfonos inteligentes y dispositivos sensoriales (como Apple Health Kit y Google Fit) con myCircadianClock. También tiene la opción de permitir que la aplicación myCircadianClock acceda a su ubicación para determinar la hora local. Puede permitir o

HP-00110284 UM IRB Approval Date 1/24/2025
Do Not Sign this Form after this Date 8/22/2025

denegar estas funciones cuando instala la aplicación myCircadianClock por primera vez, y puede cambiar estos ajustes en cualquier momento.

  - myCircadianClock tiene la opción de agregar cierta información que requiere una breve tarea fuera del teléfono inteligente, como por ejemplo: peso, estatura, circunferencia de la cintura y presión arterial. La aplicación también tiene campos opcionales para introducir resultados relevantes de análisis de sangre y muestras de orina, como: glucemia, perfil lipídico (colesterol total, LDL, HDL y triglicéridos), hemoglobina A1c, fibrinógeno, proteína C reactiva, homocisteína y cuerpos cetónicos si desea controlar estos parámetros. La introducción de estos datos es opcional.
  - <u>Todos los datos introducidos se utilizan exclusivamente para fines de seguimiento, no para diagnóstico</u>. El Instituto Salk de Estudios Biológicos, el Dr. Satchidananda Panda, y su laboratorio no son responsables de proporcionar asesoramiento médico y no son responsables de su atención médica. Debe comunicarse con un profesional médico para recibir asesoramiento médico.
  - La aplicación myCircadianClock puede proporcionar retroalimentación personalizada en forma de gráficos y texto para mostrar su progreso, y ofrecer perspectivas sobre sus comportamientos de salud.
  - La aplicación puede resumir los datos sobre cómo los patrones de alimentación, sueño o actividad en una hora específica del día están relacionados con su salud y bienestar. Estos datos pueden ayudarle a comprender mejor sus comportamientos relacionados con la salud y a manejarla mejor. Ver los gráficos y el texto es opcional, pero puede serle útil o interesante.
  - Para que la detección pasiva de su actividad física en algunos modelos de teléfono sea precisa, es importante llevar el teléfono inteligente (p. ej., en el bolsillo o la cintura). Llevar el teléfono también le ayudará a registrar los datos de alimentos, bebidas, agua y actividad tan pronto como se produzcan estos eventos.
  - En su perfil de myCircadianClock puede configurar recordatorios para que lleve a cabo las actividades de la aplicación. En general, entre más datos se introduzcan en la aplicación se producirá información más precisa e informativa.
- **Comidas, actividad y sueño:** La aplicación para teléfonos inteligentes myCircadianClock servirá como diario electrónico de alimentación, actividad y sueño.
  - En el lado del servidor, se creará un panel de control de subestudios para este proyecto específico. Los coordinadores clínicos del equipo de investigación de la Dra. Amber Kleckner tendrán acceso protegido con contraseña a los datos del estudio. En el panel de resumen del estudio, se mostrarán su código del estudio y la fecha de activación de la aplicación junto con su registro diario. Si no registra datos de alimentos durante más de 1 día, el panel de control le recordará y enviará una alerta al coordinador. Los coordinadores iniciarán sesión en el panel de control al menos dos veces a la semana para controlar los datos sobre el consumo de alimentos y hacerle el seguimiento necesario.
  - Si le asignan al grupo de alimentación con límite de tiempo, tendrá que elegir un intervalo de 10 horas al día para consumir la comida. Puede hacer seguimiento fácil de su progreso

HP-00110284 UM IRB Approval Date 1/24/2025
Do Not Sign this Form after this Date 8/22/2025

en el patrón de alimentación diario con la función de registro de tiempo de la aplicación que ofrece un resumen visual.

- Si tiene alguna dificultad para registrar datos o preguntas sobre cualquiera de las características de la aplicación, puede comunicarse con el coordinador del estudio a través de la función de comentarios de la aplicación. Las preguntas se enviarán a un servidor de correo electrónico conforme a HIPAA configurado específicamente para este estudio.

HP-00110284 UM IRB Approval Date 1/24/2025
Do Not Sign this Form after this Date 8/22/2025

CONSENTIMIENTO PARA PARTICIPAR – PÁGINA DE FIRMAS

La firma de este formulario de consentimiento indica que lo ha leído (o se lo han leído), que sus preguntas fueron respondidas satisfactoriamente y que acepta participar voluntariamente en este estudio de investigación. Recibirá un ejemplar de este formulario de consentimiento firmado.

PARTICIPANTE DE LA INVESTIGACIÓN

Si acepta participar en este estudio, firme a continuación.

Firma del sujeto	Fecha	Nombre en letra de imprenta

INVESTIGADOR O PERSONA DESIGNADA QUE OBTIENE EL CONSENTIMIENTO

Firma del investigador o designado	Fecha	Nombre en letra de imprenta

HP-00110284 UM IRB Approval Date 1/24/2025
Do Not Sign this Form after this Date 8/22/2025

LEY DE TRANSFERENCIA Y RESPONSABILIDAD DE SEGURO MÉDICO (HIPAA)
AUTORIZACIÓN PARA OBTENER, UTILIZAR Y DIVULGAR
INFORMACIÓN MÉDICA PROTEGIDA PARA INVESTIGACIÓN

Nombre del sujeto del estudio: ____

Fecha de nacimiento: ________________ **Número de historia clínica: _____________________**

NOMBRE DE ESTE ESTUDIO DE INVESTIGACIÓN: *Tome las riendas: Efectos de la periodización de nutrientes sobre la fatiga relacionada con el cáncer en los supervivientes de un cáncer de la sangre*

NÚMERO DE APROBACIÓN DEL CRI DE LA UMB: *HP-00110284*

NOMBRE DEL INVESTIGADOR: *Amber Kleckner, PhD*

INFORMACIÓN DE CONTACTO DEL INVESTIGADOR: *Department of Pain and Translational Symptom Science*
University of Maryland School of Nursing (SON)
655 W. Lombard Ave., Room 735
Baltimore MD 20201
410-706-5961

Este estudio de investigación utilizará información médica que lo identifica. Si acepta participar, este investigador utilizará únicamente la información médica que se describe a continuación.

INFORMACIÓN MÉDICA ESPECÍFICA QUE SE UTILIZARÁ Y COMPARTIRÁ:

- Datos demográficos (p. ej., estatura, peso, edad, raza, grupo étnico, educación y estado civil)
- Detalles sobre el diagnóstico y el tratamiento del cáncer (p. ej., localización del cáncer, estadio del cáncer, tipo de quimioterapia y posología, intervenciones quirúrgicas o tratamiento hormonal)
- Características clínicas (p. ej., estado menopáusico actual o estado funcional de Karnofsky)
- Análisis de sangre más reciente (p. ej., hemoglobina, hematocrito, linfocitos, etc.)
- Antecedentes médicos (p. ej., infarto de miocardio previo o diabetes).

Las leyes federales obligan al investigador a proteger la privacidad de esta información médica. La compartirá únicamente con las personas y los grupos aquí descritos.

PERSONAS Y ORGANIZACIONES QUE UTILIZARÁN O COMPARTIRÁN ESTA INFORMACIÓN:

- Dra. Amber Kleckner y el equipo del estudio.
- El patrocinador del estudio o sus representantes, como por ejemplo, los bancos de datos o las organizaciones de investigación por contrato.
- Organizaciones que coordinarán la facturación de la atención médica o el cumplimiento, como las oficinas de la Facultad de Enfermería de la Universidad de Maryland; la Universidad de Maryland, Baltimore (UMB); University of Maryland Faculty Physicians, Inc. (FPI) y

HP-00110284 UM IRB Approval Date 1/24/2025
Do Not Sign this Form after this Date 8/22/2025

consultorios del personal académico de la UMB, y el Sistema Médico de la Universidad de Maryland (UMMS).

ESTA AUTORIZACIÓN NO CADUCA, PERO PUEDE REVOCARLA EN CUALQUIER MOMENTO.

Para revocar esta autorización, envíe una carta al investigador para expresar su decisión. El investigador dejará de recopilar información médica sobre usted. Es posible que el investigador no le permita continuar en el estudio. El investigador puede utilizar o compartir información médica ya recabada.

INFORMACIÓN ADICIONAL:

- Puede negarse a firmar este formulario. Si no lo firma, no podrá participar en este estudio. Esto no afectará la atención que reciba en:
    - University of Maryland Faculty Physicians, Inc. (FPI)
    - Sistema Médico de la Universidad de Maryland (UMMS)

  Tampoco sufrirá pérdida de los beneficios que le corresponden de cualquier manera.
- En ocasiones, los organismos gubernamentales, como la Administración de Alimentos y Medicamentos o el Departamento de Servicios Sociales, solicitan copias de la información médica. Las leyes pueden exigir al investigador, a la UMSOM, FPI o UMMS que se la entreguen.
- El investigador tomará las medidas razonables para proteger su información médica. Sin embargo, las leyes federales de protección pueden no aplicarse a personas o grupos que se encuentren fuera de la UMSOM, UMB, FPI o UMMS.
- A excepción de ciertos casos especiales, tiene derecho a recibir una copia de su información médica creada durante este estudio de investigación. Puede que tenga que esperar hasta que el estudio finalice. Pregunte al investigador cómo obtener una copia de esta información del estudio.

Mi firma indica que autorizo el uso y la divulgación de mi información médica protegida para los fines descritos anteriormente. También permito que mis médicos y otros profesionales de salud compartan mi información médica protegida con el investigador para los fines descritos anteriormente.

Firma del sujeto Fecha Nombre en letra de imprenta

¿Tiene preguntas sobre la privacidad? Llame al responsable de privacidad de la UMSOM (410-706-0337) si tiene preguntas sobre sus derechos y protecciones conforme a las normas de privacidad.

¿Tiene otras preguntas? Llame al investigador que figura en este formulario para hacer cualquier otra pregunta.

HP-00110284 UM IRB Approval Date 1/24/2025
Do Not Sign this Form after this Date 8/22/2025
